# Comparative fatty acid profiling of irradiated canola varieties: insights from GC-MS metabolomics and molecular docking

**DOI:** 10.1039/d6ra05833g

**Published:** 2026-08-18

**Authors:** Zohaib Younas, Ilyas Ahmad, Faiz Ullah, Noshin Ilyas, Mahmood ul Hassan, Qusai Al Abdallah, Zia ur Rehman Mashwani

**Affiliations:** a Department of Botany, PMAS Arid Agriculture University Rawalpindi 46300 Pakistan mashwani@uaar.edu.pk zohaibyounas97@gmail.com; b Department of Plant Breeding and Genetics, PMAS Arid Agriculture University Rawalpindi 46300 Pakistan; c Faculty of Agricultural Technology, Al-Ahliyya Amman University Jordan

## Abstract

Canola (*Brassica napus*) is an edible oil consumed in human diets containing a balanced ratio of monounsaturated and polyunsaturated fatty acids (FAs). Canola oil is rich in bioactive compounds and other FA derivatives, which enhance its therapeutic potential. This study examines the effects of low-dose gamma irradiation on plant growth and modification of FA profiles in canola varieties, including NARC-Sarson, Canola-Super8, Punjab canola, and Sandal canola. On exposure to irradiation, the plants showed a net decrease in plant height, 100-seed weight and pod numbers, with a dose-dependent decrease in growth. The Gy 30 irradiation treatment reduced 100-seed weight by 19% in NARC-Sarson, whereas Gy 20 increase it by 10% in Canola-Super8. Moreover, compared with untreated control, root length, protein content, and oil content increased by 45.7%, 25%, and 48.8%, respectively, across all varieties. Among all treated varieties, Canola-Super8 showed the maximum oleic acid content, which accounts for 60.6%. Linolenic acid decreased by 31.5% in NARC-Sarson, and palmitic acid decreased by 18.75% in Canola-Super8 compared with the control. A total of seventeen FA and lipid derivatives were observed in canola varieties through GC-MS analysis. Linolenic acid displayed the highest affinity (−7.2 kcal mol^−1^) toward AKT1 kinase, forming three hydrogen-bonds interactions with target protein. In comparision, Oleic acid showed a slightly lower binding affinity (−6.6 kcal mol^−1^) at active site of AKT1 kinase. The results of this study provide a novel approach for optimizing agricultural practices with health-focused outcomes.

## Abstract 2

Canola (*Brassica napus*) is an edible oil consumed in human diets containing a balanced ratio of monounsaturated and polyunsaturated fatty acids (FAs).

## Graphical abstract

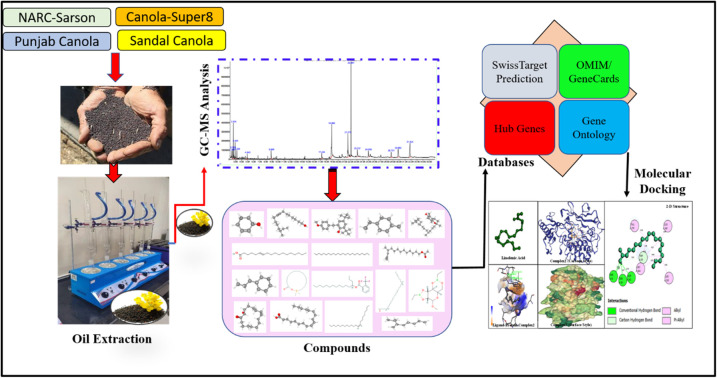

## 1 Introduction

Canola (*Brassica napus* L.) oil, a widely used edible oil, has garnered significant attention due to its balanced ratio of monounsaturated and polyunsaturated fatty acids (FAs), particularly oleic acids and α-linolenic acids.^1^ The increasing interest in this crop is largely due to its composition, comprising up to 50% fat and more than 20% protein. As a result, it serves as an indispensable resource for both oil production and animal feed industries.^2^ It serves as a valuable source of protein for both animal and human consumption while containing substantial amounts of bioactive compounds, including tocopherols, phenolic compounds, flavonoids, vitamins and phytosterols.^3^ Yates *et al.*^4^ highlighted that the bioactive compounds in canola oil enhance its therapeutic value, notably its cardiovascular benefits. Furthermore, its high-quality oil possesses health-promoting properties, such as anti-tumor, neuroprotective, antioxidant and hepatoprotective effects, rendering it valuable for human well-being.^4^

Nevertheless, external factors such as post-harvest processing methods may influence the biochemical stability and functional qualities of these FAs.^5^ Gamma irradiation, particularly with cobalt-60 (Co-60), is a well-established technology for microbial decontamination and extending the shelf life of agricultural products.^6^ Due to their strong penetrating power and lack of induced radioactivity in treated foods, cobalt-60 gamma rays are the most suitable choice for food irradiation.^7^ The sensitivity of plants to irradiation varies depending on many factors, such as species, cultivar, moisture content, ploidy level, and other physiological conditions.^8^ However, the effects of cobalt-60 gamma irradiation on structural stability and functional properties of bioactive lipids remain poorly understood.^9^ Using advanced lipidomics, we can monitor changes in the qualitative and quantitative FA composition that may be caused by radiation doses and lipid oxidation, isomerization, and saturation processes.

Dietary modifications play a crucial role in preventing cardiovascular diseases (CVDs), but evidence linking specific diets to stroke and acute myocardial infarction is limited.^10^ A report by Sargo *et al.*^11^ indicates that strokes and myocardial infarctions are the most common CVDs, accounting for nearly 80% of cardiovascular deaths. Despite significant advances in healthcare and medical interventions for cardiovascular diseases (CVDs), their global incidence remains high and substantial mortility continuing to pose major health challenges.^12^ The AKT1 protein kinase, an enzyme that regulates cell survival, metabolism, and angiogenesis, is associated with CVDs^13^ (Zhang and Guo, 2024). It has recently been discovered that specific FAs, such as omega-3 and omega-6 derivatives, can modulate AKT1 activity, making these compounds potential as therapeutic agents.^14^ Moreover, ADMET studies have been conducted simultaneously to determine the pharmacokinetics and safety profiles of these FAs, bridging the gap between agricultural science and translational pharmacology.^15^

This study reports the GC-MS identification of fatty acids in cobalt-60 gamma-irradiated canola varieties and evaluates irradiation-induced compositional variations in lipid constituents. Unlike previous studies that primarily focused on agronomic traits, the present work integrates fatty acid profiling with network pharmacology, molecular docking and ADMET prediction to computationally evaluate the potential interactions and pharmacological characteristics of selected fatty acid compounds. By integrating irradiation-induced lipid compositional changes with computational analysis targeting AKT1, this work extends conventional compositional studies to encompass a broader understanding of potential molecular associations and provides a basis for future validation studies.

## 2 Materials and methods

### 2.1 Seed collection and experimental layout

Seeds of four *Brassica napus* L. varieties (NARC-Sarson, Canola-Super8, Punjab canola, and Sandal canola) were collected from the Plant Genetic Resources Institute (PGRI) at the National Agricultural Research Center (NARC), Islamabad. Twenty-gram seed samples of each variety were irradiated at the pre-sowing stage using a gamma cell 220 irradiator with cobalt-60 doses of 10 Gy, 20 Gy, and 30 Gy, while the untreated seeds served as controls (Table 1). Irradiation was administered at a source-to-sample distance of 12 cm using facilities provided by the Nuclear Medicine Oncology and Radiotherapy Institute (NORI) at the Atomic Energy Cancer Hospital (AEAH), Islamabad. The field experiments were conducted in triplicate, with plants spaced 30 cm apart in five-meter-long rows. No fertilizer was applied during the trial, and irrigation was provided only during the germination, seedling establishment, and vegetative growth phases. Growth parameters were recorded at maturity, while seeds collected from shattered pods were dried for subsequent fatty acid profiling.

**Table 1:** Experimental design highlighting four *Brassica napus* varieties subjected to cobalt-60 irradiation treatments with different dose concentrations

Varieties	Gy 0			Gy 10			Gy 20			Gy 30		
NARC-Sarson	R1	R2	R3	R1	R2	R3	R1	R2	R3	R1	R2	R3
Canola-Super8	R1	R2	R3	R1	R2	R3	R1	R2	R3	R1	R2	R3
Punjab canola	R1	R2	R3	R1	R2	R3	R1	R2	R3	R1	R2	R3
Sandal canola	R1	R2	R3	R1	R2	R3	R1	R2	R3	R1	R2	R3

### 2.2 Morphological and yield parameters

Various morphological and yield parameters were assessed at the maturity/fruit setting stage. Plant height and root length recorded (in cm) were measured across irradiation treatments in triplicate. The average number of pods per plants was quantified, and the 100-seed weight (g) was determined for each experimental group.

### 2.3 Quantification of fatty acid profiling

The seeds collected from the pods were shade-dried and ground into a fine powder. Thirty grams of the powdered seeds were weighed, packed in filter paper, and loaded into a Soxhlet apparatus. The oil content was quantified by soaking each sample in 200 mL of hexane for 6 hours using the Soxhlet extraction method. The fatty acid profiles of the extracted oil seeds were analyzed following the protocol described by Aram *et al.*^16^ with minor modifications. Gas Chromatography-Mass Spectroscopy (GC-MS; Unicam 4600 system) was employed for the analysis, with the chromatographic data processed using Hunter Workstation software (ver. B.04.00). The electron ionization source was operated at 70 eV, and separation was achieved using an OPTIMA-5 capillary column (30 m × 0.22 mm, 0.25 µm film thickness). The detector temperatures were maintained at 180 °C for the column, 240 °C for the injection site, and 280 °C for the detector. Helium served as the carrier gas at a flow rate of 1 mL min^−1^, and a 20 µL sample was injected using an automated liquid sampler.

### 2.4 ADMET screening of fatty acids (FAs)

A total of 17 fatty acids (FAs) and their derivatives were selected to evaluate their efficacy, and their performance was compared with that of the reference drug rosuvastatin (compound CID: 446157) against CVD-associated targets (SI). All these FA compounds were subjected to the PubChem database (https://pubchem.ncbi.nlm.nih.gov/) to obtain isomeric SMILES, which is mandatory for absorption, distribution, metabolism, excretion, and toxicity (ADMET) screening. Further, these ligands were analyzed using *in silico* tool ADMETlab 3.0 (https://admetlab3.scbdd.com/) to obtain the pharmacokinetic properties of the potential drug targets. The web server, SwissADME (https://www.swissadme.ch/index.php), was used to predict the oral bioavailability scores of the selected compounds at a threshold of BA > 0.55.^17^ Then, the compounds were screened for drug-likeness scores using druglikeFilter 1.0 (https://drugfilter.idrblab.cn/process_filter_), and compounds that fulfilled the selection criteria threshold with QED > 0.15 were used for further computational analysis.^18^ According to Lipinski's rule of five (RO5), the MW should be <500 Da, TPSA should be <120 Å^2^, HBA/HBD <10 and <5, log *P* should be ≤ 5, and no more than 10 rotatable bonds should be present.^19,20^

### 2.5 SwissTargetPrediction

The SMILES representation of the selected ligands/query molecules was entered into the SwissTargetPrediction (https://www.swisstargetprediction.ch/) online server for the retrieval of compound targets (gene symbols and UniProt IDs) along with target classifications.^21^ Furthermore, potential targets associated with cardiovascular disease (CVD) were systematically retrieved from Online Mendelian Inheritance in Man (OMIM) database (https://mirror.omim.org) and the Human GeneCards (https://www.genecards.org/) databases by searching the keyword ‘‘cardiovascular disease’’. A Venn diagram was constructed using the Venny 2.1 online tool to obtain an overlapping analysis of genes for compound-disease targets, which enables the construction of PPI networks based on node number and other topological parameters using the STRING database (ver. 12.0) and Cytoscape (v3.10.3).^22^

### 2.6 Selection of ligand molecules and protein selection

Prior to the docking analysis, the input SMILES of the selected ligand molecules was entered into the PubChem database, and a 3D conformer was saved in a structure data file (SDF) format, which was later converted to a PDBQT file.^23^ The crystal structure of AKT1 kinase complexed with allosteric inhibitor 27 was obtained from RCSB Protein Data Bank (https://www.rcsb.org/) with (PDB ID: 6HHG) and downloaded in the PDB format for subsequent molecular docking analysis.^24^

### 2.7 Protein preparation and molecular docking

During protein preparation, natural ligands, heteroatoms, and water molecules were removed, and polar hydrogen atoms were subsequently added, whereas the targets were saved in PDB format. Ligand-receptor interactions were visualized using the BIOVIA Discovery Studio visualizer. Molecular docking was performed to determine the binding affinity, orientation preferences, and molecular interactions of bioactive metabolites from *Brassica napus* with target protein associated with CVD *i.e.*, AKT1 kinase AutoDock Vina (v. 1.5.7) software was used for molecular docking analysis. Finally, ligand-protein complexes were displayed in both 2-D and 3-D representations, with results documented as PNG images.^25^ The PyRx Virtual screening software (version 0.8) was applied for re-docking purposes to verify the accuracy of the *in silico* data based on the binding affinity scores and RMSD scores.^26^ Parameters of the grid box were defined for docking purposes, with the center *X*: 1437, *Y*: −13.34, and *Z*: −13.98, and dimensions (angstroms) *X*: 40.917, *Y*: 56.23 and *Z*: 52.11.

### 2.8 Statistical analysis

Morphological and yield parameters were analyzed using a two-factor randomized complete block design (RCBD) in Minitab 18.1 and SPSS was used to calculate the mean values and standard deviations from triplicate measurements. Significant differences among canola varieties were assessed using *F*-statistics and *p*-values. Graphical representations were generated using PAST software and GraphPad Prism (v. 9.5). Venn diagrams illustrating metabolite-disease target overlaps were constructed using the online tool Venny 2.1.

## 3 Results

### 3.1 Cobalt-60 irradiation effects on growth parameters and fatty acid profiling

**Fig. 1:**
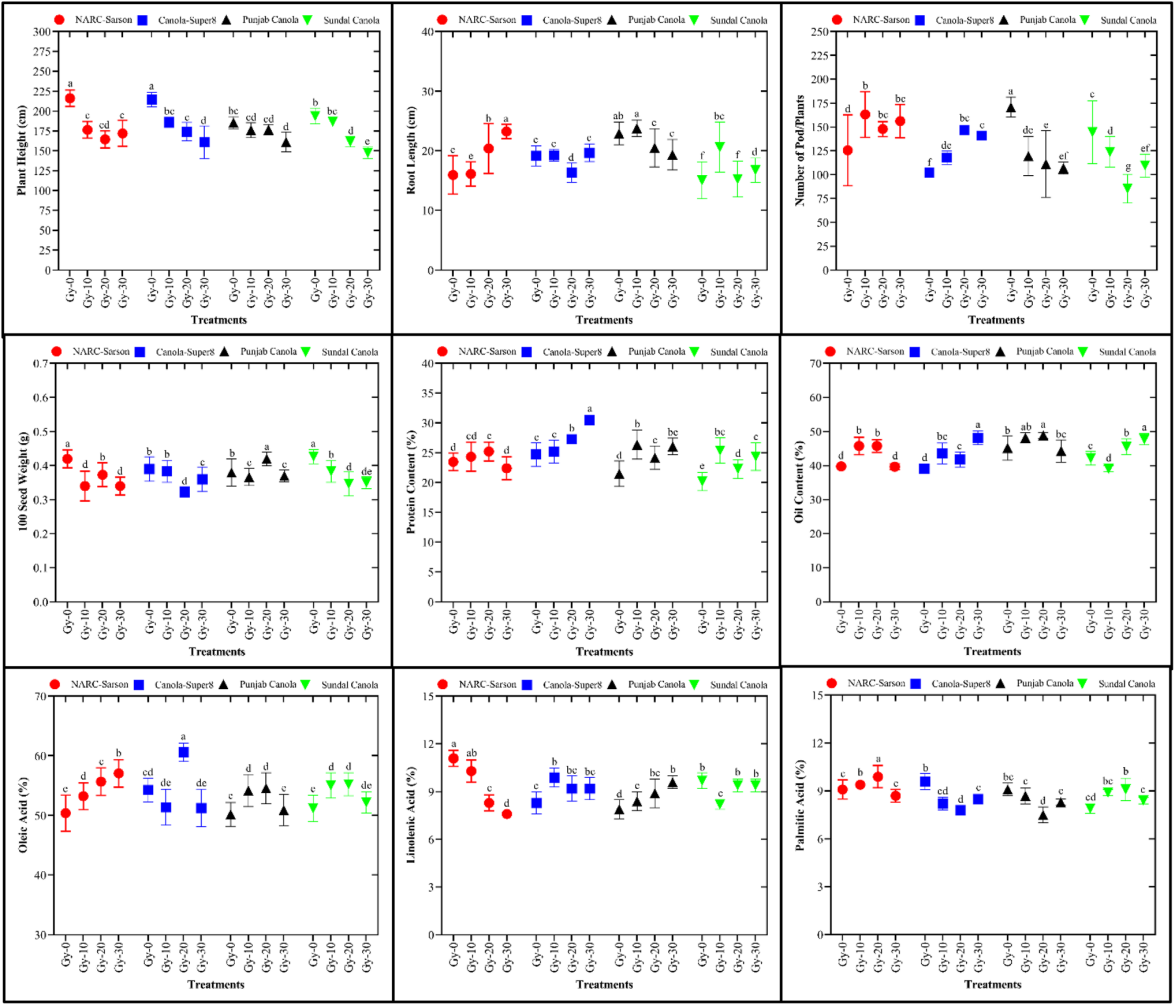
Impact of cobalt-60 gamma irradiation on plant growth metrics and fatty acid profiles of *Brassica napus* varieties. A multiple bar graph was generated from the mean values ± S.D. Letters on the bar graph indicate differences in irradiation doses across *Brassica napus* varieties.

The present study evaluated the effects of cobalt-60 irradiation on the growth parameters and fatty acid profiles of four *Brassica napus* varieties. As shown in Fig. 1, untreated NARC-Sarson plants exhibited the greatest average plant height (216.47 ± 10.39 cm), followed by Punjab canola (185.63 ± 7.57 cm). In contrast, plants exposed to cobalt-60 gamma irradiation showed a dose-dependent reduction in plant height, with higher irradiation doses negatively affecting growth. At the highest dose (Gy 30), Sandal canola and Canola-Super8 recorded the lowest mean heights (147.73 ± 7.08 cm and 161.00 cm, respectively). ANOVA (supplementary material, Table 1) indicated statistically significant results for variety (*F* = 3.09; *p* = 0.041), irradiation (*F* = 33.11; *p* = 1.198 × 10^−9^), and variety × irradiation interaction (*F* = 2.44; *p* = 0.031). Punjab canola exhibited the greatest mean root length (23.80 ± 1.4 cm) under the Gy 10 irradiation treatments, indicating a potentially stimulatory effect at moderate irradiation levels. At Gy 30, NARC Sarson and Canola-Super8 recorded root lengths of 23.27 ± 1.19 cm and 19.70 cm, respectively, whereas Sandal canola showed a maximum root length of 20.63 ± 4.2 cm. Notably, the Gy 30 dose increased root length relative to the control by 45.7% in NARC-Sarson, 11% in Sandal canola and 2.7% in Canola-Super8, suggesting genotype-dependent variability in irradiation response, as shown in Fig. 1. However, ANOVA (SI, Table S2) reveals a significant variation in canola varieties (*F* = 7.154; *p* = 0.0009), and the variety × irradiation interaction is also significant (*F* = 3.44; *p* = 0.005), whereas the main effect of irradiation alone is not significant (*F* = 1.731; *p* = 0.181), implying that cobalt-60 gamma irradiation did not exert a consistent effect on root elongation across the tested *Brassica napus* varieties. Fig. 1 shows that cobalt-60 irradiation increased pod numbers in NARC-Sarson and Canola-Super8, whereas it reduced pod numbers in Punjab canola and Sandal canola. ANOVA revealed that the irradiation main effect is not significant (*F* = 1.119; *p* = 0.356), so irradiation levels do not show a consistent overall difference when averaged across varieties. The variety main effect is significant (*F* = 6.95; *p* = 0.001), whereas the variety × irradiation interaction is highly significant (*F* = 6.572; *p* = 0.0001) in the pod number analysis, indicating that the effect of irradiation depends on variety. Notably, CanolaSuper8 exhibited a 43.6% increase at Gy 20, while NARC-Sarson showed a 29.9% increase at Gy 10 compared with the control (untreated plants). In contrast, the Gy 20 dose reduced the pod number in the Sandal and Punjab canola varieties, highlighting the genotype-specific response to irradiation, as depicted in Fig. 1. In this study, a reduction in seed weight was observed across different *Brassica napus* varieties following irradiation. Untreated plants exhibited the highest 100-seed weight, whereas irradiated plants generally showed decreased values. At Gy 30, seed weight declined by 19% in NARC-Sarson and 17.9% in Sandal canola compared with that of the control. Interestingly, the Gy 20 irradiation dose induced a 10% increase in 100-seed weight in Canola-Super8, as shown in Fig. 1. ANOVA demonstrated that the main effect of variety was statistically non-significant (SI, Table S4), whereas irradiation treatment and variety × irradiation interaction were highly significant (*p* < 0.05), indicating that the response of different varieties varied markedly at different irradiation levels.

The results demonstrated that gamma irradiation significantly enhanced the protein content across all tested canola cultivars. A low dose (Gy 10) increased the protein content by 25% in Sandal canola and 22.7% in Punjab canola, whereas a moderate dose (Gy 20) increased the protein content by 7% in NARC-Sarson. The greatest enhancement (23.1%) was observed in Canola-Super8 at Gy-30 (Fig. 1). The dose-dependent variations highlight genotype-specific responses to irradiation. A detailed statistical analysis revealed significant effects of variety (*F* = 11.96; *p* = 0.0001), irradiation (*F* = 9.57; *p* = 0.0001), and their interaction (*F* = 3.53; *p* = 0.004) on the protein percentage (SI, Table S5). Gamma irradiation influenced the oil content across four different *Brassica napus* varieties, with the highest yields observed at 20 Gy. At this dose level, Punjab canola and NARC-Sarson exhibited oil contents of 48.9% and 45.8%, respectively, while Sandal canola and Canola-Super8 showed 47.8% and 48.2%, respectively, compared with their untreated controls at Gy 0. Notably, untreated Canola-Super8 and NARC-Sarson recorded the lowest oil contents (39.1% and 39.8%, respectively). ANOVA shows that the results are significant for variety (*F* = 8.26; *p* = 0.004) and irradiation treatment (*F* = 8.19; *p* = 0.004), while the effects of interactions on the oil percentage are highly significant (*p* < 0.0001) (SI, Table S6). Fig. 1 shows significant irradiation-induced differences in oleic acid content among the *Brassica napus* varieties (*F* = 5.60; *p* < 0.05). Further, the statistical results indicate that variety and interaction effects are not significant at *p* = 0.05. At Gy 20, Canola-Super8 exhibited the highest oleic acid percentage (60.6%), followed by Sandal canola (55.6%), compared with the untreated controls. Untreated plants had the lowest oleic acid levels, with Canola-Super8 and NARC-Sarson at 50.2% and 50.8%, respectively (Fig. 1). In contrast, ANOVA revealed that the main effects of variety (*F* = 2.580; *p* < 0.05) and irradiation (*F* = 0.909; *p* = 0.447) were not significant for linolenic acid content across the four *Brassica napus* varieties, whereas the variety × irradiation interaction (*F* = 11.84; *p* < 0.001) was significant, indicating variations in linolenic acid content across the four *Brassica napus* varieties. At Gy 30, the linoleic acid content was reduced by 31.5% in NARC-Sarson and 3% in Sandal canola compared to that in untreated plants (Fig. 1). Conversely, the high irradiation dose markedly increased linolenic acid levels by 21.5% in Punjab canola and 10% in Canola-Super8, highlighting the genotype-dependent divergence in fatty acid metabolism under radiation stress. ANOVA reveals no statistically significant differences in palmitic acid content among the four *Brassica napus* varieties (NARC-Sarson, Canola-Super8, Punjab canola, and Sandal canola) under irradiation treatments (*F* = 2.74; *p* < 0.059) and interaction effect (*F* = 7.80; *p* < 0.0001), while the variety effect was significant (*F* = 10.12; *p* < 0.0001). However, at the Gy 20 irradiation dose, palmitic acid levels decreased by 18.75% in Canola-Super8 and 17.58% in Punjab canola compared with the controls, whereas Sandal canola and NARC-Sarson exhibited increases of 15.1% and 8.7%, respectively (Fig. 1). The divergent responses emphasize the genotype-specific modulation of fatty acid metabolism under cobalt-60 gamma irradiation.

### 3.2 Physico-chemical and ADMET screening of fatty acid compounds

Absorption, distribution, metabolism, excretion, and toxicity (ADMET) screening is a critical step in drug discovery and development. It evaluates the pharmacokinetic behaviour and safety profiles of potential drug candidates early in the process, helping to identify promising compounds while eliminating those likely to fail in later stages. The first step in drug discovery is to assess the physico-chemical properties of candidate compounds. The key parameters include log *S*, log *P*, log *D*, topological polar surface area (TPSA), boiling point, melting point, p*K*_a_ (acid and base), compound density, van der Waals volume, and hydrogen bonding capacity (both donors and acceptors), as well as the stretching potential of fatty acid (FA) compounds. A detailed list of the physico-chemical properties of the FA compounds is provided in SI (Table S1). In ADMET screening, caco-2 permeability indicates how well a compound can cross biological membranes, such as the intestinal absorption or the blood–brain barrier. Negative values suggest poor permeability, and good oral candidates typically have log Pe values greater than −5.0.

**Fig. 2:**
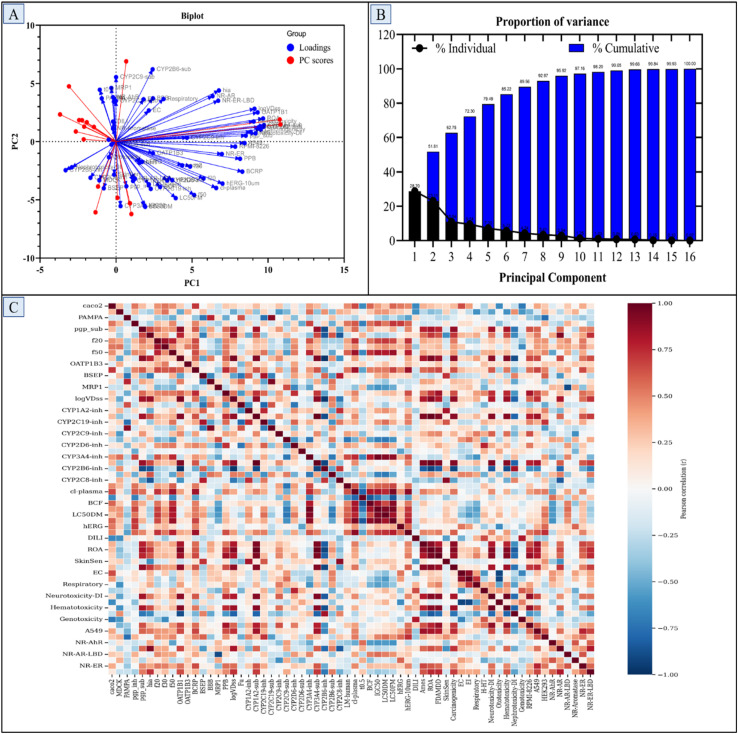
(A) Principal component analysis (PCA) of the ADMET properties of fatty acid (FA) compounds identified in *Brassica napus*, (B) proportion of variance explained by principal components (PCs), illustrating both the individual percentage contribution of each PC and the corresponding cumulative percentage of total variance, and (C) heatmap showing the correlation of all ADMET variables.

In this study, GC-MS analysis of *Brassica napus* varieties identified 17 fatty acids (FAs) and lipid derivatives. The simplified molecular input line entry system (SMILES) of these selected compounds was retrieved from the PubChem database and evaluated for absorption, distribution, metabolism, excretion, and toxicity (ADMET) properties using ADMETlab 3.0. Quantitative data comprising 64 variables (SI) were obtained from ADMETlab 3.0 (online web server) and analyzed using multivariate analysis, including principal component analysis (PCA) and heatmap correlation. Principal component analysis (PCA) revealed that the 16 principal components (PCs) accounted for most of the variance, illustrating the distribution pattern of variables across the coordinate space (Fig. 2A and PC scores illustrated in the SI). By reducing the dimensionality of the datasets, PCA captures the majority of the variance (85.22% cumulative variance for PC1 to PC6) into fewer orthogonal axes. The PCA of the ADMET data revealed a cumulative value of 51.81% for the first two principal components (PC1 and PC2), with eigenvalues of 18.37 and 14.79, respectively. The loading scores indicated that PC1 was primarily influenced by LC_50_FM (0.681) and IGC_50_ (0.192) while PC2 was associated with log VDss (0.519), PBB (0.388) and HIA (0.211), as presented in the supplementary material. Principal component 3 (PC3) was influenced by the CYP2C19 inhibitor (0.270) and CYP2D6 inhibitor (0.298), accounting for 10.94% of the explained variance, with an eigenvalue of 7.002, as shown in Fig. 2B. Principal component 4 (PC4) was associated with the CYP2C9 inhibitor (0.354), Fu (0.227), and CYP2D6 substrate (0.109), contributing 8.54% of the variance (Fig. 2B) and exhibiting an eigenvalue of 6.108, as shown in the SI. These four principal components (PCs) collectively simplify datasets into interpretable patterns, highlighting the key drivers of variability in *Brassica napus* compounds, such as bioavailability, toxicity and metabolic pathways. Moreover, the P-gp inhibitor, CYP2C9-inhibitor, EC, and H-HT exhibited the highest loading scores in PC5, which accounted for 7.20% of the total variance, with an eigenvalue of 4.605. In contrast, BSEP, CYPIA2-inhibitor, and CYP29-substrate predominately contributed to PC6, explaining 5.73% of the total variance. PC7–PC16 is provided in the SI, showing their variance and eigenvalues for each principal component (PC), while loading scores demonstrated that the key variables influenced each particular principal component in the PCA analysis. Fig. 2C depicts the variable correlation through a heatmap, where red indicates a positive correlation and blue represents a negative correlation. A strong positive correlation (*r* = 0.71) was observed between CaCO-2 permeability and BCRP activity, followed by a moderate correlation with P-gp inhibitor (*r* = 0.56). A weak positive correlation (*r* = 0.31) was observed between CaCO-2 permeability and MDCK permeability. The absorption parameter MDCK exhibited a weak negative correlation (*r* = −0.31) with the distribution marker MRP1. CYP isoforms (1A2, 2C19, 2C9, and 2D6) emerged as critical biomarkers, with their inhibitors and substrates reflecting metabolic risk factors in living organisms. A negative correlation was identified between CYP2C9-substrate (*r* = −0.22), CYP26D-substrate and CYP2B6-inhibitors with key parameters such as BBB penetration, plasma protein binding (PPB), and fraction unbound (Fu). Fig. 2B illustrates the comprehensive correlation patterns among the ADMET properties, highlighting the interactions that are critical for drug availability and safety profiling.

### 3.3 SwissTargetPrediction for *Brassica napus* fatty acids (FAs)

**Fig. 3:**
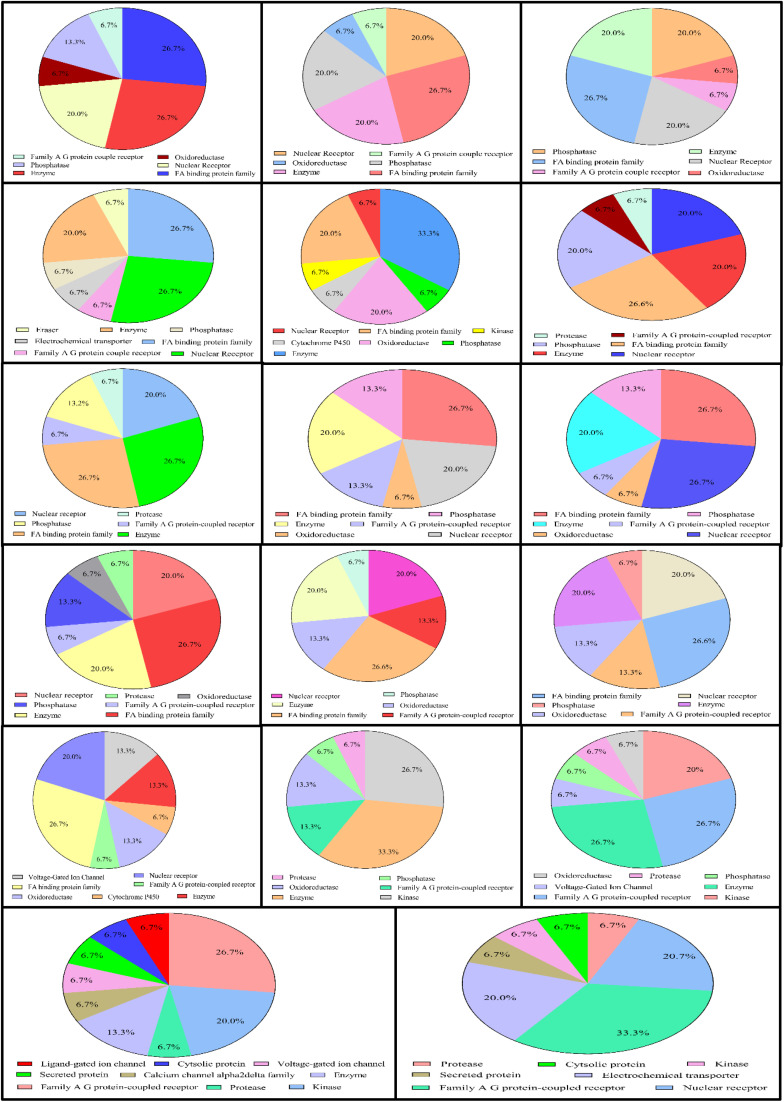
Pie chart displaying the percentage distribution patterns of the targeted classes of *Brassica napus* FAs.

The targets for candidate compounds or metabolites were identified by submitting the isomeric SMILES of fatty acids (FAs) and lipid derivatives to SwissTargetPrediction, which generated standardized gene names and classified the predicted targets, as shown in Fig. 3. All fatty acids (FAs) predominately interacted with the targeted classes, showing the highest percentage distributions, including the fatty acid binding protein family, electrochemical transporters, enzymes, erasers, nuclear receptors, cytochrome P450, kinase, protease, voltage-gated ion channel, secreted proteins, ligand-gated ion channel, calcium channel auxiliary α–δ subunit family, cytosolic proteins, and family A-G protein-coupled receptors (Fig. 3). Analysis using SwissTargetPrediction, combined with Venn diagram comparisons, reveals a significant overlap between plant metabolites, such as fatty acids (FAs) and cardiovascular-disease-related targets, highlighting their therapeutic potential. The top 15 target classes for the 17 fatty acids (FAs) were identified, and their relative contributions were visualized in a pie chart, as shown in Fig. 3. Oleic acid predominately influenced six targeted classes: adipocyte fatty acid-binding proteins, enzymes, nuclear receptors, phosphatases, oxidoreductases and family A-G protein-coupled receptors. Among these, FABP4 and enzymes accounted for 26.7% of the total class distribution. 9-Octadecanoic acid also targeted six classes, with the highest abundance observed for fatty acid-binding protein (FABP4) at 26.7%, followed by nuclear receptors (PPARG) and phosphatases (PTPN1) at 20% each. Fig. 3 visualizes the distributions using a pie chart, illustrating the relative abundance of target classes influenced by *Brassica napus* FAs.

### 3.4 Common metabolites of fatty acids and disease targets

Two databases, the OMIM database and GeneCards, were used to retrieve genes associated with cardiovascular disease (CVD), yielding a total of 16483 targets. Predicted metabolite targets for all fatty acids (FAs) were obtained from SwissTargetPrediction, resulting in 1700 genes (SI).

**Fig. 4:**
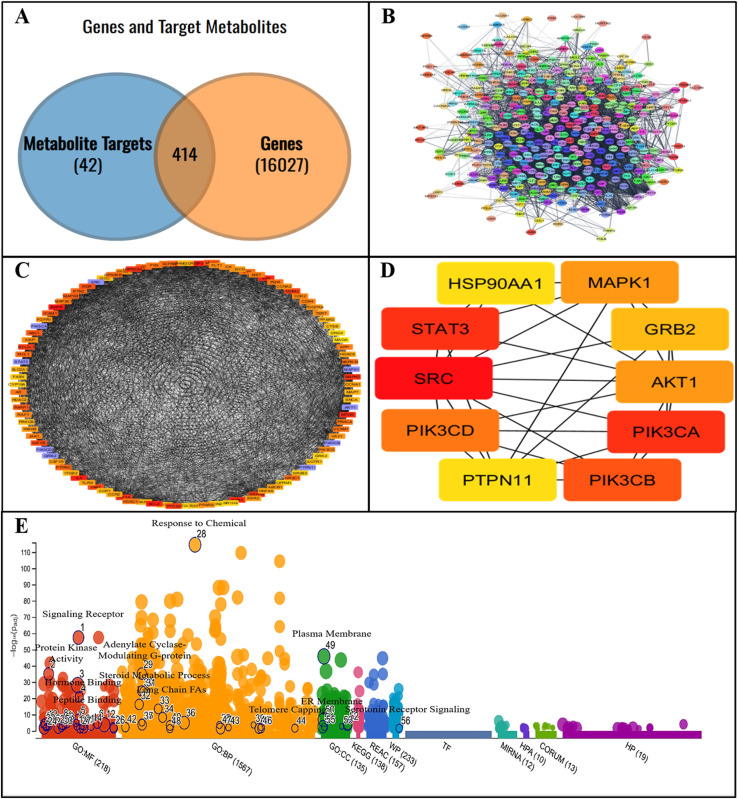
(A) Venn diagram illustrating the number of overlapping genes of fatty acids (FAs) and lipid derivatives derived from *Brassica napus* and cardiovascular disease, (B) protein–protein interaction (PPI) network of the overlapping targets to evaluate functional connectivity, (C) circular network visualization of the top 100 ranked genes, highlighting the hub genes in color target *via* Cytoscape, (D) identification of top 10 predictive hub genes associated with cardiovascular disease (CVD) based on degree centrality method, and (E) gene ontology (GO) enrichment analysis for the overlapping genes, summarizing their involvement in various target classes and enrichment pathways.

These potential targets were assigned probability scores and annotated with the corresponding gene names and UniProt IDs, enabling their classification into specific target classes. A Venn diagram analysis comparing cardiovascular disease (CVD)-related targets and fatty acids and their derivatives or metabolite-associated targets identified 414 overlapping genes. A detailed assessment revealed that *Brassica napus* had 414 common targets with cardiovascular disease (CVD), and the remaining 16027 CVD-related genes were exclusively associated with CVD (Fig. 4A). These overlapping targets include key genes and proteins involved in critical cardiovascular-related pathways, such as lipid metabolism, fatty acid biosynthesis, substrate-related disorders, carcinoma-associated pathways, vascular diseases and many others.

### 3.5 Identification of hub genes and gene ontology

**Fig. 5:**
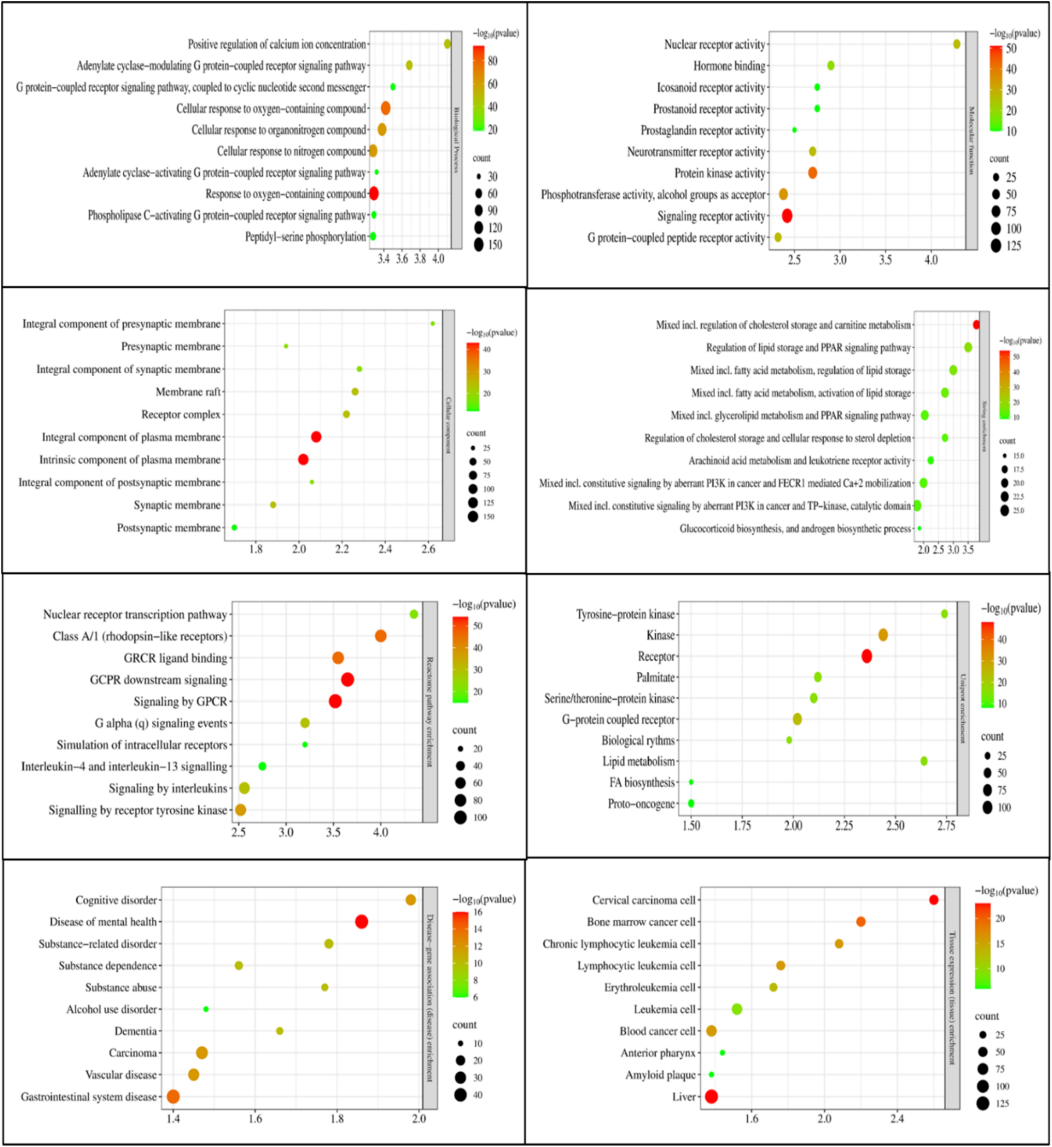
Bubble plot representing gene ontology (GO) enrichment analysis with respect to biological processes, molecular functions, cellular components, STRING enrichment, Reactome pathway enrichment, UniProt enrichment, disease–gene association (disease) enrichment, and tissue expression profiling. The top 10 predictive targets and pathways associated with overlapping genes were highlighted based on gene count and –log 10 (*p*-value), where the bubble size represents the gene count and the bubble color intensity corresponds to the enrichment significance.

A protein–protein interaction (PPI) network of the common targets was constructed using the STRING database, where nodes represent proteins and edges denote functional associations. The network was generated using STRING 12.0 and visualized using Cytoscape. Hub genes were identified using the degree centrality method, revealing 10 highly connected genes strongly associated with cardiovascular disease (CVD) and involved in the co-expression and functional regulation of proteins linked to cancer, inflammatory disorders, and cardiovascular diseases. Among the 414 common targets, the top 100 genes ranked by network connectivity were initially selected, from which the top 10 hub genes were prioritized based on their high degree values (highlighted in Fig. 4B and C). The overall PPI network comprised 414 nodes and 2757 edges, with a statistically significant enrichment *p*-value (<0.0001) and a clustering coefficient of 0.466, indicating a non-random and biologically meaningful interaction network. Further analysis identified a sub-network consisting of the top 10 hub genes, interconnected by 27 edges and exhibiting a significant *p*-value of 0.0055. The subnetwork showed an average node degree of 6.75 and a high clustering coefficient (0.964), reflecting strong interconnectivity among these critical genes (Fig. 5D). The large number of common gene and protein targets between plant metabolites and CVD-associated genes highlights their potential as natural therapeutic agents, offering a robust scientific basis for further investigation of their role in CVD management. Gene ontology (GO) enrichment analysis demonstrated that these common targets are predominately involved in key molecular functions and biological processes, including modulating G protein–protein receptor, receptor-mediated signaling, response to chemical stimuli, protein kinase activity, steroid metabolic process, long chain fatty acids, telomere capping, peptide binding, hormone binding, catalytic activity, ion binding, and serotonin receptor signaling (Fig. 4E).

Biological process (gene ontology, GO) annotations indicated that the predictive overlapping genes for FA and lipid derivatives are involved in modulating cellular responses to oxygen- and nitrogen-containing compounds, lipid metabolism, peptide and hormone signalling, and inflammatory pathways. As illustrated in Fig. 5, fatty acids (FAs) exhibited limited gene involvement in G-protein coupled receptor (GPCR) pathways despite exhibiting an exceptionally low false discovery rate (FDR = 1.0 × 10^−19^), indicating high statistical significance. In contrast, processes like ‘‘response to oxygen-containing compounds’’ showed a comparatively higher FDR value (1.0 × 10^−78^), suggesting lower confidence in their functional enrichment. Overall, GO enrichment analysis identified 2172 terms within the biological process category, 269 terms within molecular functions and 162 terms within cellular components (Fig. 5). Within the molecular function category, 150 genes were associated with signaling receptor activity, representing the highest contribution, whereas 10 genes were linked to prostaglandin receptor activity, representing the lowest level of enrichment. Gene Ontology (GO) enrichment analysis of cellular components revealed that 100 out of 162 genes were significantly associated with the intrinsic and integral components of the plasma membrane. Enrichment analysis of Reactome pathways revealed that 110 genes are critically involved in GPCR signalling and receptor downregulation pathways, suggesting that membrane-associated signal transduction is a central regulatory mechanism. Moreover, disease–gene association analysis demonstrated strong enrichment in mental health disorders, gastrointestinal diseases, carcinoma and cardiovascular diseases, with the highest gene representation observed in these categories. Consistently, tissue-specific expression profiling showed predominant enrichment in the liver (150 genes) and cervical carcinoma cells (50 genes), as shown in Fig. 5.

### 3.6 Molecular docking

**Fig. 6:**
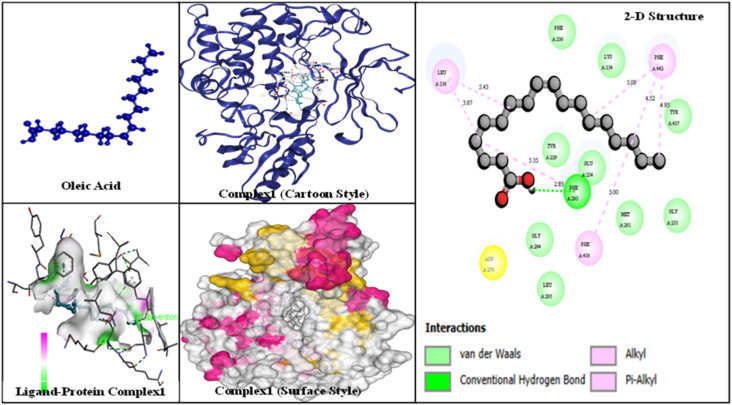
2D-3D structures of the oleic acid-6HHG complexes. The figure shows a 3D structure, a cartoon view of the complex, a surface structure and a 2D overview of the oleic acid-6HHG complex showing the interacting residues and the bonding pattern.

Molecular docking analysis was performed to explore the potential interactions between the selected fatty acids and cardiovascular disease (CVD)-associated protein structures (PDB: 6HHG). The docking results suggest that oleic acid forms a stable ligand-protein complex with 6HHG through a hydrogen bond involving the PHE A:293 residue at a distance of 2.83 Å. The docking analysis revealed additional π–π stacking interactions involving PHE A:442, LEU A:156, and PHE A:438, which may contribute to ligand stabilization within the binding pockets. Furthermore, van der Waals interactions was observed with LYS A:154, TYR A:229, GLY A:233, GLU A:234, PHE A:236, MET A:291, GLY A:294, LEU A:295, PHE A:293, and TYR A:437 (Fig. 6). The top three docked poses of the oleic acid-6HHG complex exhibited binding affinities of −6.6 kcal mol^−1^, −5.5 kcal mol^−1^, and −4.7 kcal mol^−1^ (Table 2), indicating a moderate predicted binding strength under *in-silico* conditions.

**Table 2:** Docking scores of fatty acid (FA)-6HHG protein associated with CVDs

Fatty acid biomarker (ligand)	Conformational poses	RMSD low bound–RMSD upper bound	Docking score (kcal mol^−1^)	Bonding interaction	Amino acids
Oleic acid	1	0–0	−6.6	Van der Waals, H-bonding, alkyl/π–alkyl	PHE442, LEU156, PHE438, ASN279, GLY294, LEU295, PHE293, TYR229, GLU234, MET291, GLY233, TYR437, LYS154, PHE236
	2	1.505–2.494	−5.4		
	3	2.678–6.312	−4.7		
Linolenic acid	1	0–0	−7.2	H-Bonding, C–H bonding, alkyl/π–alkyl	VAL270, LYS268, LEU210, LEU268, TRP80, THR81, THR82, ASP292
	2	1.344–2.823	−6.1		
	3	1.703–2.152	−4.8		
Palmitic acid	1	0–0	−6.3	H-Bonding, pi-sigma, C–H bonding, alkyl/π–alkyl, unfavorable donor–donor	LEU264, TRP80, TYR272, VAL270, LYS268, LEU210, THR82
	2	1.515–2.29	−6.2		
	3	1.582–2.595	−6.0		
Rosuvastatin	1	0–0	−9.7	H-Bonding, pi–sigma, pi–cation, C–H bonding	ASP32, GLU49, LEU52, GLY327, ARG328, ALA329, LYS389, GLY394
	2	0.198–2.301	−7.3		
	3	3.169–4.941	−6.4		

**Fig. 7:**
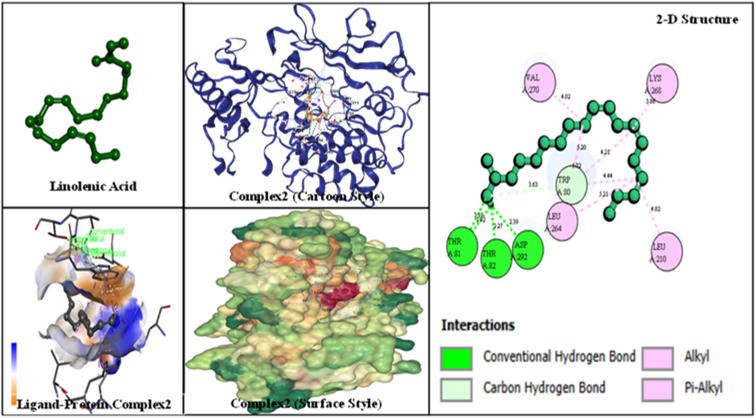
Docked linolenic acid-GHHG complexes presented in the 3D structure, 2D structure, cartoon view and surface view. The 2D structure displays the bonding pattern present in the residue interactions of the ligand with the target protein.

Linolenic acid demonstrated comparatively stronger predicted binding affinities of −7.2 kcal mol^−1^, −6.1 kcal mol^−1^, and −4.8 kcal mol^−1^ for its top three poses. The interaction profile revealed three hydrogen bonds involving THR A:81, THR A:82 and ASP A:292 within the active site of 6HHG (Fig. 7). Additionally, alkyl and π–alkyl interactions were observed at LEU A:210, LEU A:264, LYS A:268, and VAL A:270, with intermolecular distances ranging from 3.88 Å to 5.21 Å. Although weaker pi–pi stacking interactions were detected than those of oleic acid, the presence of multiple hydrogen bonds and favourable binding energies suggests a potentially stable interaction pattern in docking studies.

**Fig. 8:**
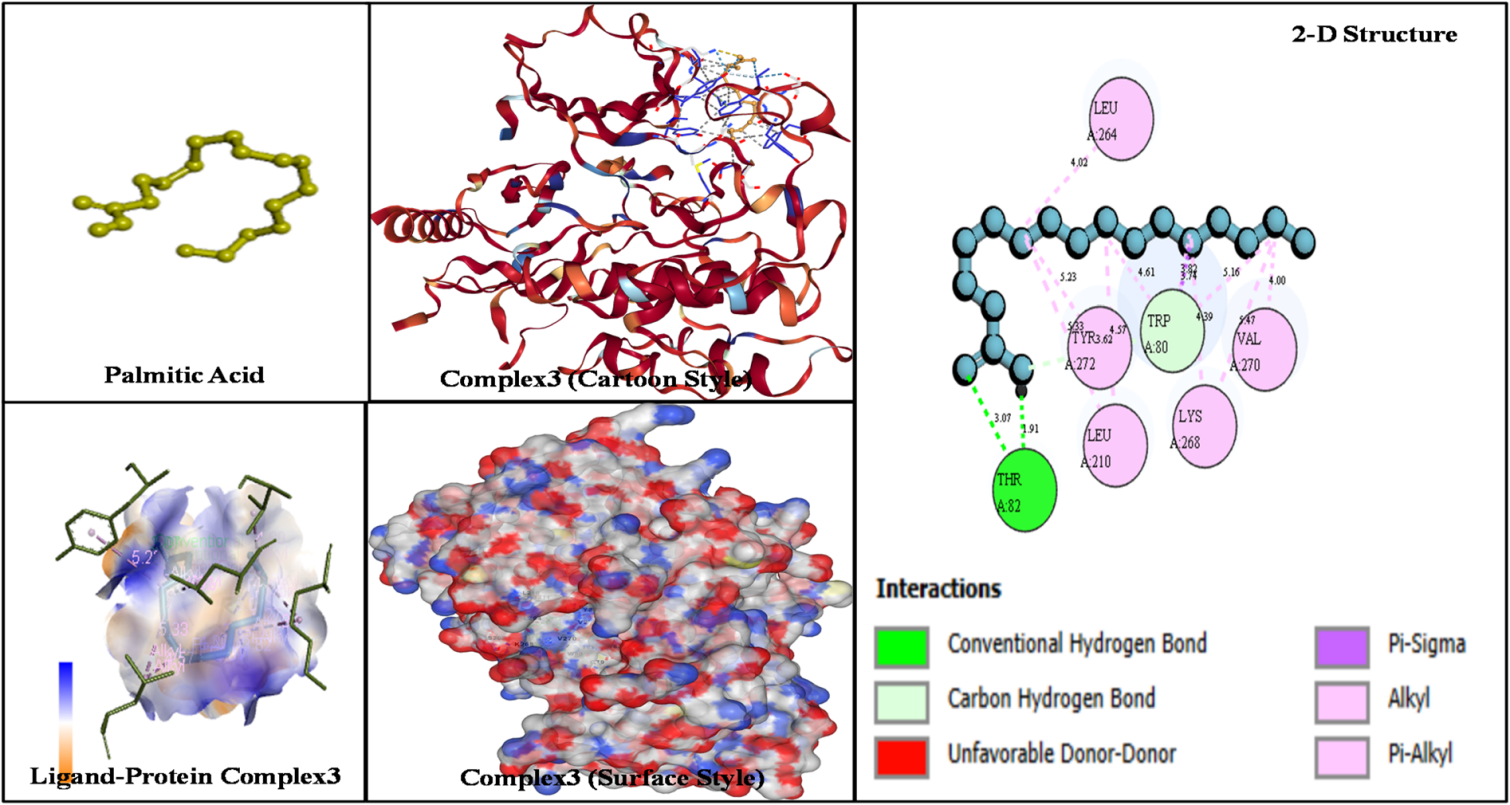
Docked complexes of palmitic acid-6HHG protein. The figure consists of the ligand molecule, 3D structure of the ligand–protein complex, cartoon view, surface view and 2D view of the complex.

For palmitic acid, docking studies indicated strong interactions with TRP A:80, THR A:82, LEU A:210, LEU A:264, VAL A:270, LYS A:268, and TYR A:272 within the predicted active site of the protein associated with CVD (Fig. 8). A hydrogen bond with THR A: 82 at a distance of 3.01 Å and a carbon–hydrogen bond involving TRP A:80 were observed. Additional π–π stacking and hydrophobic interactions contributed to ligand stabilization, with intermolecular distances ranging from 2.8 to 5.33 Å. The calculated binding affinities for the top three docked poses were −6.3 kcal mol^−1^, −5 kcal mol^−1^, and −4.5 kcal mol^−1^ (Table 2), indicating a comparatively lower binding strength than linolenic acid.

**Fig. 9:**
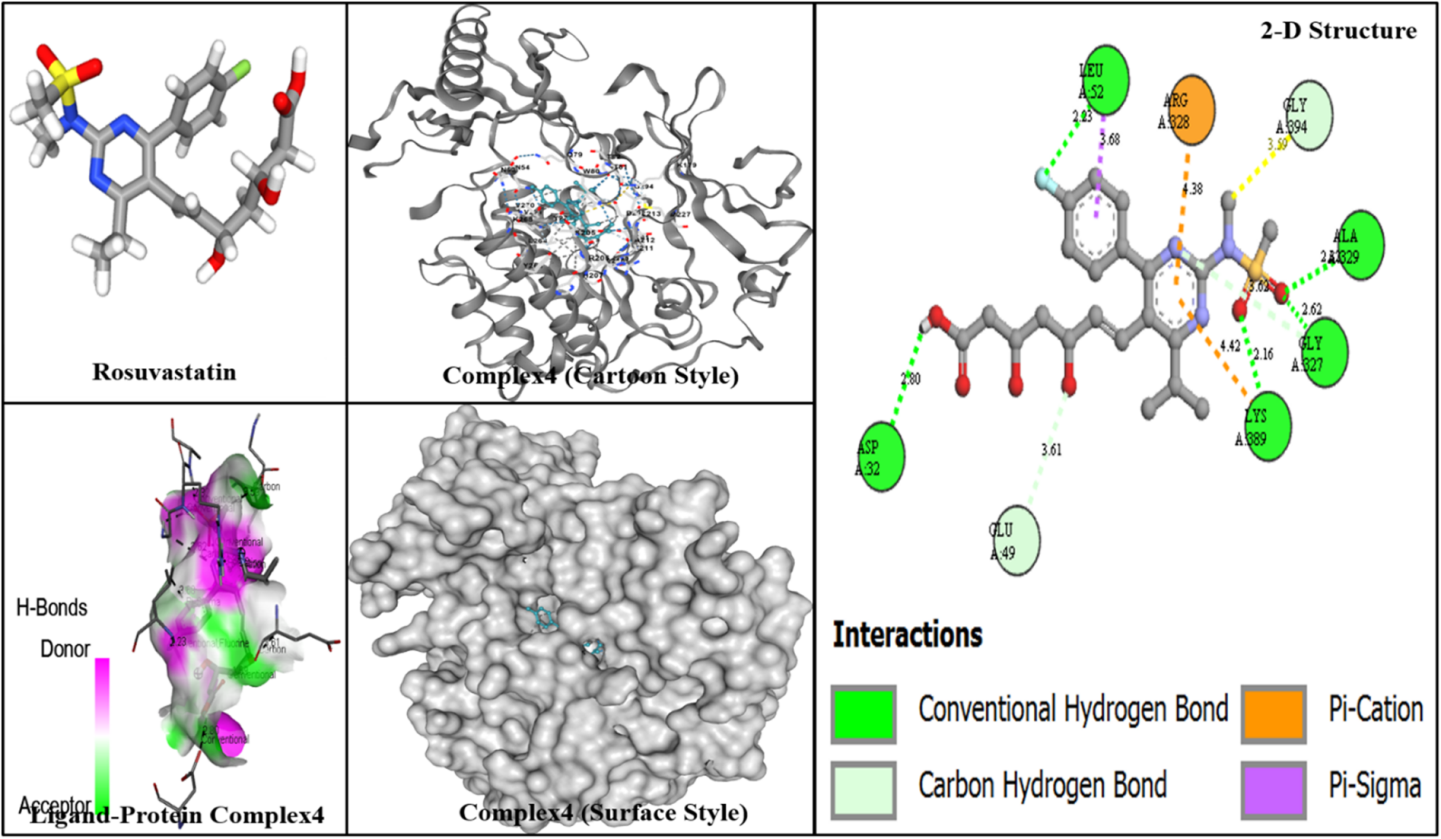
2D and 3D structures of the docked complex of the rosuvastatin-6HHG protein. The control drug (rosuvastatin) exhibited strong residue interactions with the active protein sites.

Rosuvastatin formed five hydrogen-bonds with AKT1 kinase (PDB ID: 6HHG) protein contributing to stability of protein-ligand complex. The three binding poses exhibited favourable binding affinities of −9.3, −7.3 and −6.4 kcal mol^−1^, respectively. The intermolecular distances range from 2.16 to 4.42 Å among the residue interactions, which involve interactions at ASP32, GLU49, LEU52, GLY327, ARG328, ALA329, LYS389, and GLY394 (Fig. 9). Overall, the docking results suggest that linolenic acid may exhibit a stronger *in silico* binding affinity toward the 6HHG protein associated with cardiovascular disease (CVD) compared to oleic acid and palmitic acid. However, these findings are based solely on computational modelling and represent predictive interactions. Experimental validation through *in vitro* and *in vivo* studies is required to confirm the biological relevance of ligand–protein interactions and their potential implications in cardiovascular disease pathways.

## 4 Discussion

*Brassica napus* holds economic significance due to its oil, which is abundant in polyunsaturated fatty acids and bioactive compounds.^27^ However, the current study demonstrated that cobalt-60 doses led to a gradual decrease in plant height and seed-related characteristics, suggesting a mostly inhibitory reaction at moderate to elevated exposure levels. These constituents make it a nutritionally robust alternative to olive oil.^28^ All parts of *Brassica napus* have industrial and nutritional applications; however, the seeds are the most economically important component as a major source of edible oil and protein 2. Canola oil extraction also generates substantial by-products (40 million tons per year), underscoring its global agro-industrial significance.^29^ Given its polyunsaturated fatty acid (PUFA) content, particularly omega-3 and omega-6, canola oil aligns with dietary recommendations that advocate replacing saturated fatty acids to reduce cardiovascular risks.^30^ Comparable dose-dependent growth suppression in canola varieties has been reported by Shah *et al.*,^31^ who observed reduced plant height and pod number, whereas Rahimi and Bahrani^32^ reported declines in plant height and seed weight beyond 100 Gy in *Brassica napus* varieties. Likewise, Parthasarathi *et al.*^33^ reported growth inhibition in sesame at 250–450 Gy. Conversely, other crops exhibit stimulatory or biphasic responses. Shabani *et al.*^34^ reported 3–19% increases in wheat height at 100–200 Gy, while Iqbal *et al.*^35^ observed enhanced soybean growth at 200 Gy, followed by a decline at higher doses. Rajeev *et al.*^36^ further demonstrated genotype-dependent variations in sunflower mutants, affecting flowering and oil traits. Franco *et al.*,^37^ consistent with Luckey^38^ threshold hypothesis (100 Gy), described low dose stimulation of germination and productivity, while inhibitory effects exceeded this limit. Beyond this threshold, the inhibitory trends observed in our study reduced plant height and seed weight in canola, increasing under cobalt-60 doses, which is consistent with the findings in the sesame varieties. The present study demonstrated a dose-dependant response in the agronomic traits of the *Brassica napus* varieties, with low-dose irradiation promoting growth and higher irradiation doses adversely affecting these traits. Therefore, the response of *Brassica napus* appears to be strongly dose- and genotype-dependent, emphasizing the necessity of precision irradiation optimization to balance mutagenic benefits with agronomic stability.

Previous studies have suggested that the effects of gamma irradiation on Brassica species are highly dose-dependent and genotype-specific. Khan *et al.*^39^ showed that siliqua or pod numbers were increased at 10 krad (187 siliqua per plant) compared to the control (174 siliqua per plant), whereas high doses (15–30 krad) drastically decreased pod numbers. Likewise, excessive irradiation may impair reproductive structures, as reported by Marcheva *et al.*,^40^ where irradiated canola mutants exhibited poor siliqua and reduced yields. Comparing the results of previous studies with those of the current study showed an increase in pod number among *Brassica napus* varieties at a low dose of irradiation (Gy 10), while a decline in pod number at high doses Gy 20 and Gy 30 suggests that the threshold for beneficial stimulation was exceeded, leading to cumulative cellular damage. At higher doses, excessive reactive oxygen species production likely disrupts meristematic activity, impairs floral differentiation, and induces chromosomal aberration, thereby reducing the pod set. Likewise, Anwar *et al.*^41^ and Ebrahimi *et al.*^42^ noticed that low doses of gamma irradiation significantly enhance the protein content in Brassica napus (canola). In this study, an increase in protein content was observed, consistent with previous findings. Rahimi and Bahrani^32^ observed improvements in oil content and fatty acid composition (oleic acid and linolenic acid) up to 200 Gy, followed by declines at higher doses. Another study conducted by Anwar *et al.*^41^ reported no significant overall alteration in fatty acid profiles, although oleic acid increased with irradiation dose, while linolenic acid showed a non-linear response. Mutation-induced alterations in fatty acid composition have also been documented in *Brassica napus* and sunflower by Bhatia *et al.*,^43^ supporting the role of gamma irradiation in modifying the key enzymatic steps of lipid metabolism. In the present study, higher doses of gamma irradiation (Gy 20 and Gy 30) enhance the fatty acid composition, particularly oleic acid, linolenic acid and palmitic acid. Ahire^44^ reported metabolic competition within the fatty acid denaturation pathway, providing a biochemical explanation for the shifts observed in irradiated mutants. Previous studies have suggested that irradiation below 10 kGy is widely recognized for improving food shelf-life without adverse health effects;^45,46^ its impact on plant physiology remains strongly species- and dose-dependent. Collectively, these findings highlight that gamma irradiation is a useful tool for mutation breeding but requires genotype-specific calibration to avoid detrimental agronomic consequences.

**Fig. 10:**
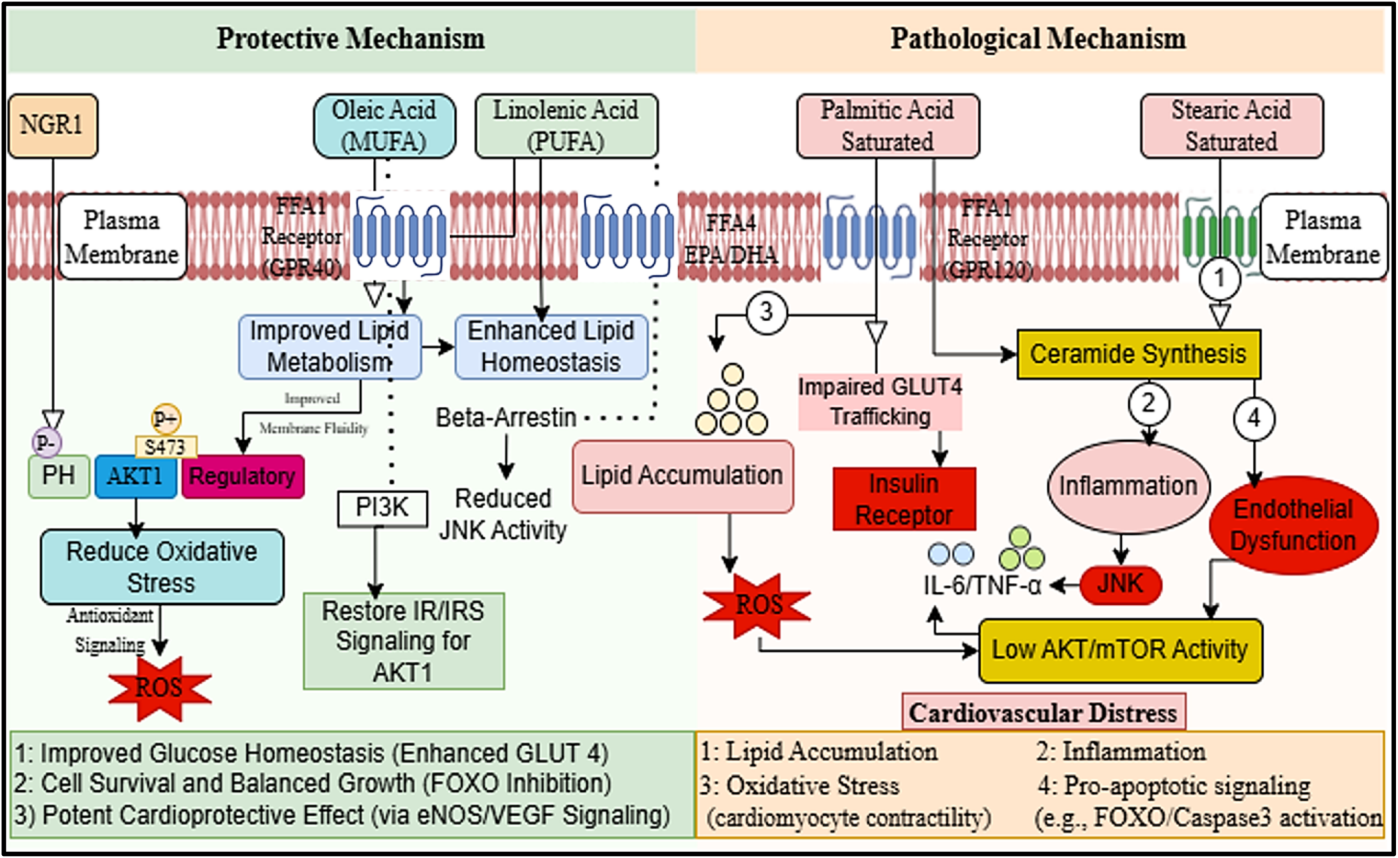
Dual metabolic effects of fatty acids, showing that saturated fatty acids induce cardiovascular distress through metabolic dysregulation and unsaturated fatty acids promote cardioprotection.

Previous metabolomic studies have demonstrated that irradiation significantly alters the chemical composition of plant-derived oils. For instance, Zhang *et al.*^47^ identified 91 volatile compounds in edible oils from sesame, peanut, and flaxseed, reporting a 14% increase in hydrocarbons, aldehydes and ketones following irradiation at 8 kGy. In comparison, the present study identified 150 compounds in irradiated *Brassica napus* (canola) oil across doses ranging from 0 to 30 Gy using GC-MS analysis. These compounds were categorized into 11 chemical groups, including 17 fatty acid and lipid derivatives, which were selected for further computational investigation. Li *et al.*,^48^ investigated overall metabolic changes and pathway enrichment following irradiation. In contrast, the present study focused especially on fatty acid related compounds and evaluated their potential biological significance based totally on using computational approaches. Gene ontology (GO) enrichment analysis was performed to explore the predicted involvement in biological processes, cellular components and molecular functions. This target strategy allowed the identification of fatty acids with potential interactions relevant to cardiovascular-associated molecular pathways. Notably, Pekkoh *et al.*^49^ suggested that oleic acid and linolenic acid may interact with the angiotensin-converting enzyme (ACE), a protein implicated in cardiovascular regulation. In the present docking analysis, linolenic acid demonstrated a comparatively favourable binding affinity toward the CVD-associated AKT1 protein under *in-silico* conditions. Given the established role of AKT1 in vascular signalling and chronic cardiovascular pathophysiology, this predicted interaction may have a potential modulatory effect. However, these findings are based solely on a computational approach and should be interpreted as hypothesis-generating rather than as confirmatory evidence of inhibition. Furthermore, ADMET prediction suggested favourable pharmacokinetic properties for linolenic acid, including acceptable absorption and distribution characteristics. Although a previous study by Xu *et al.*^50^ highlighted the therapeutic relevance of polyunsaturated fatty acids (PUFAs) in chronic inflammatory conditions, the present study demonstrates computational projections and requires experimental validation to confirm their biological efficacy. In terms of the proposed pathway, it can be suggested that the cardioprotective effect of NGR1 could occur through the modulation of lipid metabolism and the restoration of metabolic equilibrium, while saturated fatty acids cause metabolic imbalance and induce heart problems. Unsaturated fatty acids (oleic acid and linolenic acid) seem to activate free fatty acid (FFA) receptors and the PI3K/AKT1 downstream pathway, leading to improved lipid metabolism, increased membrane fluidity, and restoration of insulin receptor substrate (IR/IRS) signaling. The activation of AKT1 plays a crucial role in decreasing oxidative stress *via* antioxidant signaling, resulting in decreased ROS production and improved cellular viability. Further, the decreased activity of JNK and increased GLUT4-mediated glucose uptake act synergistically to promote lipid hemostasis and metabolic balance. In addition, an increased level of saturated fatty acids (palmitic acid and stearic acid) causes an increase in ceramide production, interferes with trafficking of GLUT4, and interrupts the signaling of the insulin receptor. These responses lead to the accumulation of lipids, excess production of ROS, release of inflammatory cytokine (IL-6/TNF-α) and activation of JNK, leading to decreased AKT/mTOR activity. These responses directly lead to endothelial dysfunction, apoptosis-related signaling and cardiovascular distress (Fig. 10).

In conclusion, this study combines metabolic profiling with network-based and molecular docking strategies to provide predictive insights into the potential biological relevance of irradiation-derived fatty acids. These results form the basis for future experimental studies to validate their roles in cardiovascular-related molecular pathways.

## 5 Conclusions

In this research, it is shown that gamma irradiation can bring about dose-dependent changes in plant growth and productivity as well as fatty acid levels. There was a significant decrease in agronomic traits at high doses of irradiation, but a few parameters were significantly improved, like root length, protein content and oil quality. Metabolomics analysis revealed differences in the levels of fatty acids such as oleic acid, linolenic acid and palmitic acid across *Brassica napus* germplasms. Similarly, this study shows predicted favorable pharmacokinetic properties for the selected fatty acids in terms of absorption and distribution parameters. The prediction of molecular docking and ADMET studies on certain fatty acids against AKT1 kinase is purely hypothetical in nature and hence must not be considered as biological effects, but simply as predictions from a computational point of view. In conclusion, gamma irradiation induced measurable metabolic changes in *Brassica napus* germplasm, particularly in its fatty acid composition. These finding highlights the potential of irradiation as a tool for generating metabolically diverse germplasm. However, further *in vitro* and *in vivo* studies are needed to determine whether these metabolic changes translate into meaningful nutraceutical or cardioprotective effects.

## Author's contribution

ZY conceptualized and drafted the original manuscript, IA conducted software implementation and data analysis, FU participated in reviewing and editing, NI contributed to formal analysis, reviewing and editing, MULH contributed to methodology and funding acquisition, QA participated in re-docking analysis, review and editing, and ZURM conceptualized the research framework, supervised the project, and critically revised the manuscript.

## Conflicts of interest

All the authors declare no competing interests.

## Supplementary Material

RA-OLF-D6RA05833G-s001

RA-OLF-D6RA05833G-s002

RA-OLF-D6RA05833G-s003

## Data Availability

All data generated or analyzed during this study are included in this published article and its supplementary information (SI). Supplementary information is available. See DOI: https://doi.org/10.1039/d6ra05833g.
