# Supplementary material for: Comparative fatty acid profiling of irradiated canola varieties: insights from GC-MS metabolomics and molecular docking
Source: RSC Adv. 2026 Aug 18;16(41):48734–50. doi: 10.1039/d6ra05833g (PMC13484239; doi:10.1039/d6ra05833g)
Supplement: Supplementary file 1 [file RA-016-D6RA05833G-s001.pdf]

This journal is © The Royal Society of Chemistry 2026

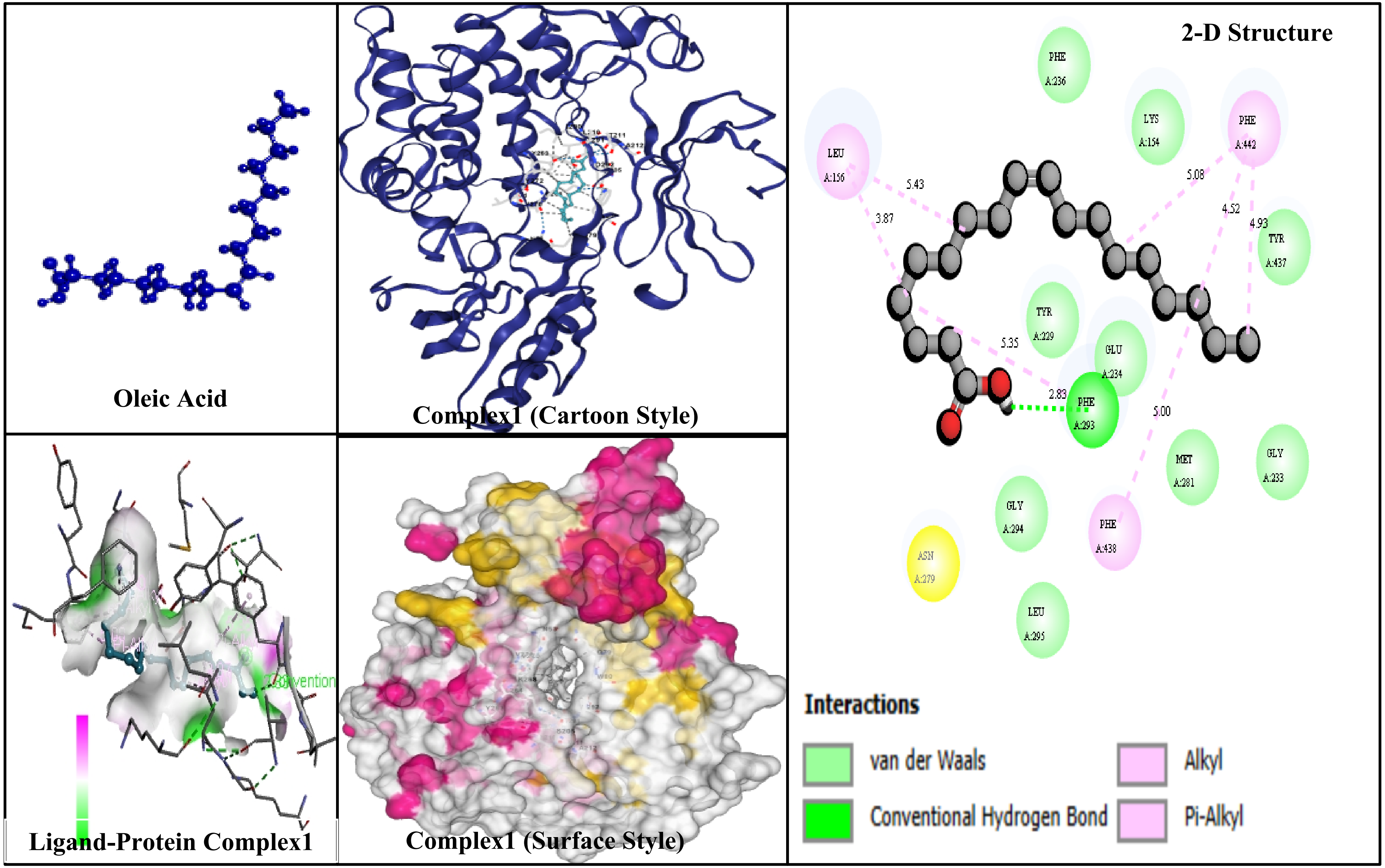

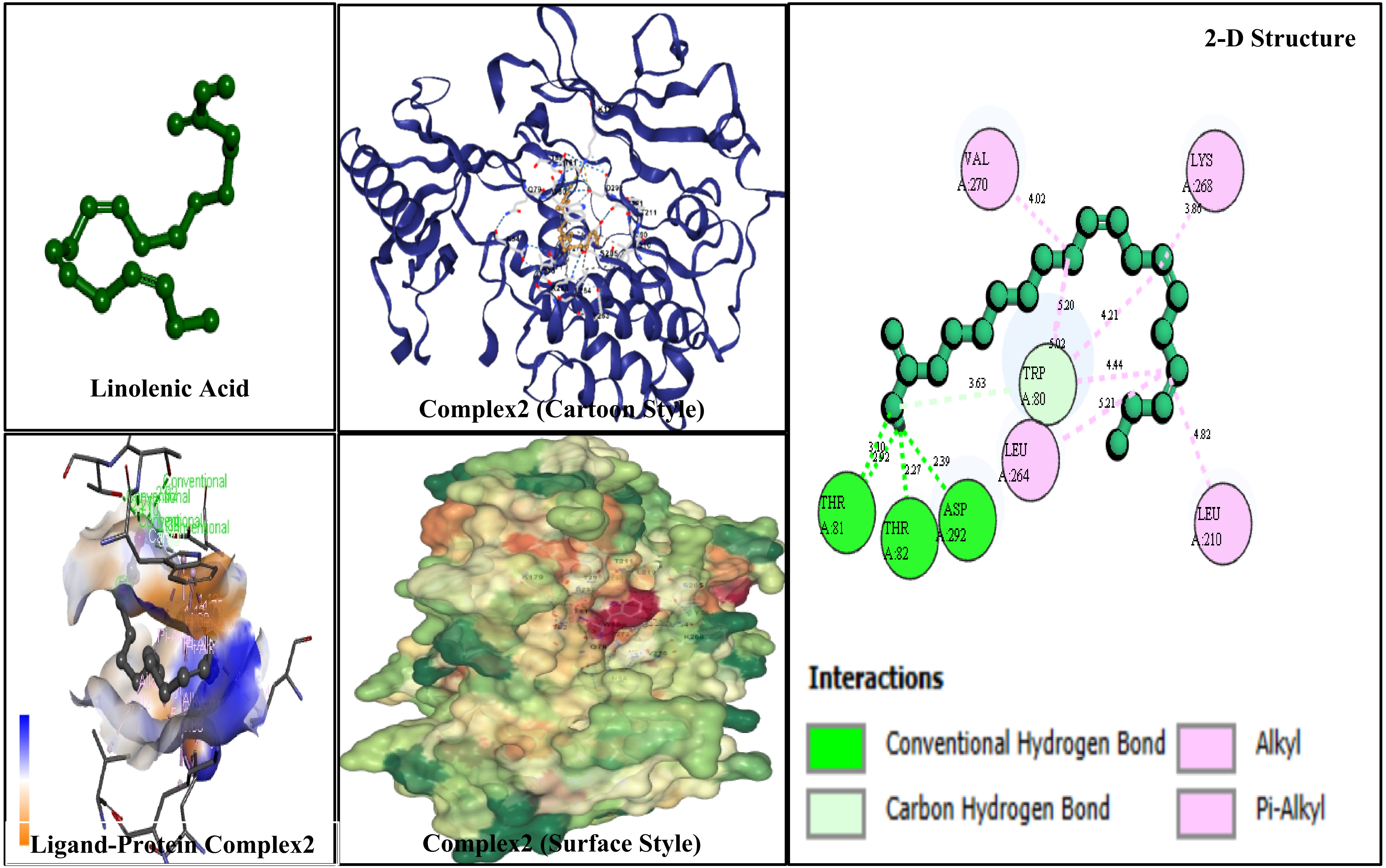
Linolenic Acid
Complex2 (Cartoon Style)
Ligand-Protein Complex2
Complex2 (Surface Style)
2-D Structure
VAL A:270
LYS A:268
TRP A:80
LEU A:264
LEU A:210
THR A:81
THR A:82
ASP A:292
4.02
3.86
5.20
4.21
5.02
4.44
3.63
5.21
4.82
3.10
2.92
2.27
2.39
Interactions
Conventional Hydrogen Bond
Carbon Hydrogen Bond
Alkyl
Pi-Alkyl

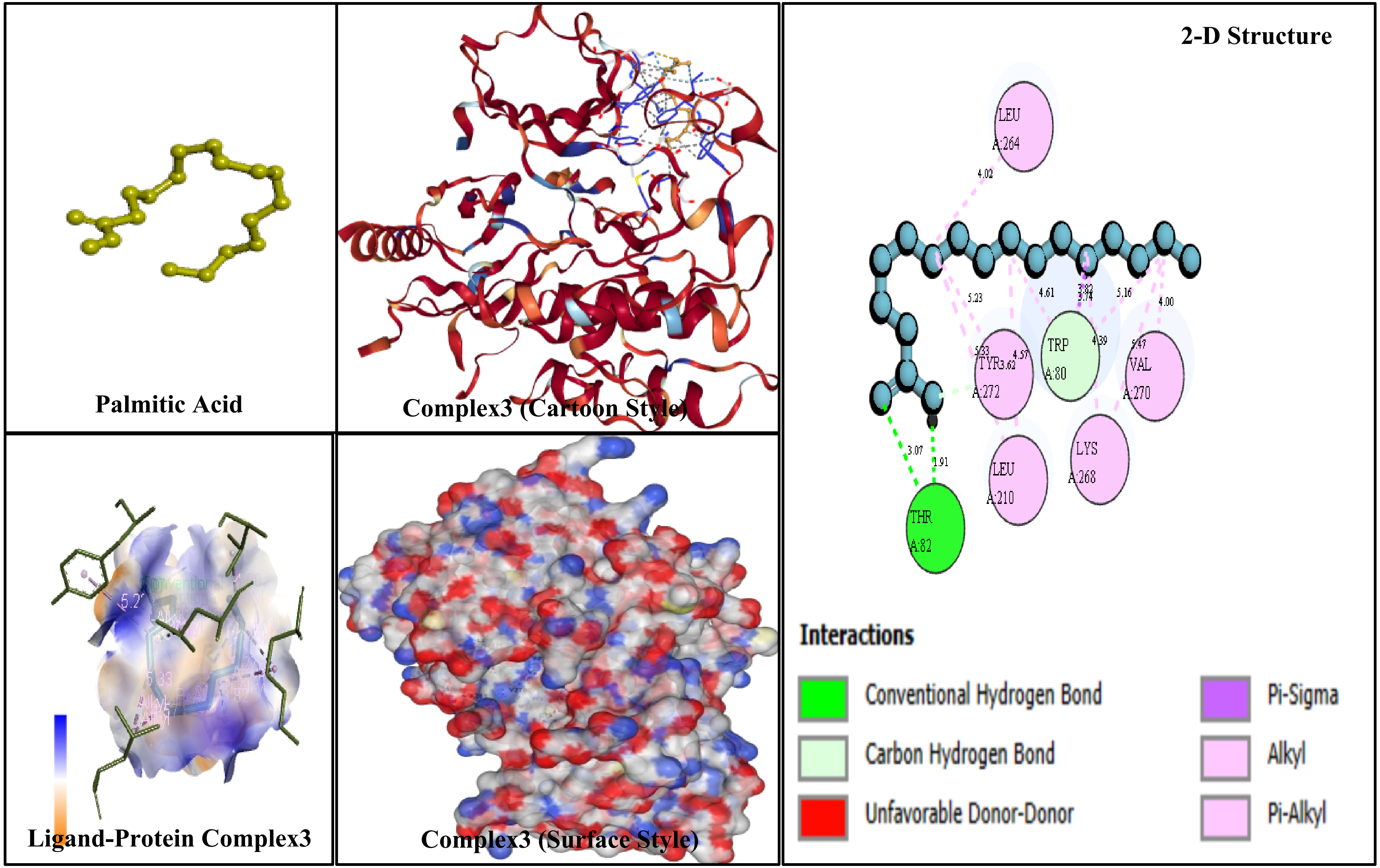
Palmitic Acid
Complex3 (Cartoon Style)
Ligand-Protein Complex3
Complex3 (Surface Style)
2-D Structure
LEU
A:264
4.02
5.23
4.61
5.16
4.00
TYR
A:272
TRP
A:80
VAL
A:270
LEU
A:210
LYS
A:268
THR
A:82
3.07
1.91
Interactions
Conventional Hydrogen Bond
Carbon Hydrogen Bond
Unfavorable Donor-Donor
Pi-Sigma
Alkyl
Pi-Alkyl

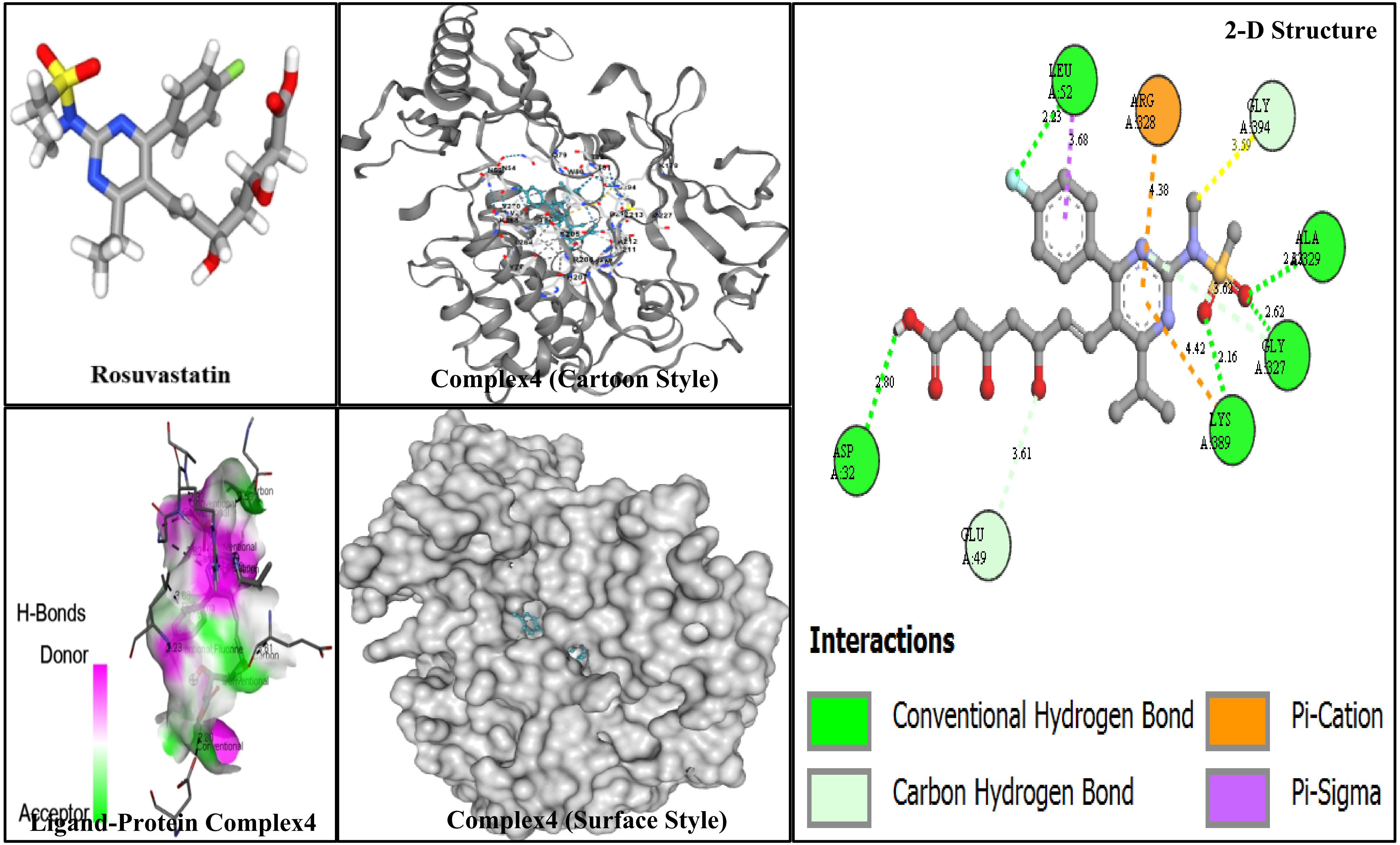
Rosuvastatin
Complex4 (Cartoon Style)
2-D Structure
LEU A:52
ARG A:328
GLY A:394
ALA A:329
GLY A:327
LYS A:389
ASP A:32
GLU A:49
2.23
3.68
4.38
3.59
3.62
2.62
4.42
2.16
2.80
3.61
Interactions
Conventional Hydrogen Bond
Pi-Cation
Carbon Hydrogen Bond
Pi-Sigma
H-Bonds
Donor
Acceptor
Ligand-Protein Complex4
Complex4 (Surface Style)
