# Supplementary material for: Comparative fatty acid profiling of irradiated canola varieties: insights from GC-MS metabolomics and molecular docking
Source: RSC Adv. 2026 Aug 18;16(41):48734–50. doi: 10.1039/d6ra05833g (PMC13484239; doi:10.1039/d6ra05833g)
Supplement: Supplementary file 2 [file RA-016-D6RA05833G-s002.pdf]

Supplementary Information (SI) for RSC Advances.
This journal is © The Royal Society of Chemistry 2026

Supplementary files

Table of content entry

Table 1: Physico-chemical properties of fatty acids (FAs) compounds identified in Brassica napus L using GC-MS analysis.

Fatty Acids	MW	Vol	Dense	nHA	nHD	TPSA	nRot	logS	logD	logP	mp	bp	pka_acidic	pka_basic
Oleic Acid	282.26	332.19	0.85	2	1	37.3	15	-5.87	3.70	7.06	1.74	352.12	5.04	2.93
9-Octadecenoic acid	282.26	332.19	0.85	2	1	37.3	15	-5.65	3.66	6.64	11.43	338.67	5.05	3.37
cis-Vaccenic acid	282.26	332.19	0.85	2	1	37.3	15	-5.91	3.72	7.09	3.07	352.51	5.06	2.96
Palmitic acid	256.24	300.24	0.85	2	1	37.3	14	-5.69	3.65	6.65	54.32	334.76	5.13	4.05
Ethyl Oleate	310.29	366.78	0.85	2	0	26.3	17	-7.42	4.33	7.63	-30.73	318.95	8.87	4.95
Paullinic acid	310.29	366.78	0.85	2	1	37.3	17	-6.77	4.01	7.88	14.61	367.76	5.30	3.35
cis-11-Eicosenoic acid (Gonodoic acid)	310.29	366.78	0.85	2	1	37.3	17	-6.73	4.00	7.84	13.03	367.48	5.28	3.31
9,12-Octadecadienoic acid (Linoleic acid)	280.24	329.56	0.85	2	1	37.3	14	-5.43	3.64	6.95	-20.35	359.23	4.91	2.23
9-Octadecenoic acid, methyl ester	296.27	349.49	0.85	2	0	26.3	16	-7.06	4.24	7.28	-9.10	313.81	8.00	4.72
Erucic acid	338.32	401.38	0.84	2	1	37.3	19	-7.56	4.31	8.60	28.15	378.18	5.69	4.13
8,11-Eicosadienoic acid	308.27	364.15	0.85	2	1	37.3	16	-5.94	3.80	7.21	-4.73	356.61	5.08	2.88
10,13-Octadecadienoic acid	280.24	329.56	0.85	2	1	37.3	14	-5.47	3.65	6.86	-5.92	355.01	4.79	2.25
9-Octadecenal	266.26	323.40	0.82	1	0	17.07	15	-6.35	3.86	6.43	2.85	289.31	6.88	5.10
2,3-Dihydroxypropyl elaidate	356.29	401.66	0.89	4	2	66.76	19	-3.74	3.64	4.89	41.24	317.27	6.86	5.83
9-Octadecenoic acid (Z)-, 2,3-dihydroxypropyl ester	356.29	401.66	0.89	4	2	66.76	19	-4.01	3.77	5.12	38.28	319.82	7.18	5.83
Palmitoyl chloride	274.21	306.66	0.89	1	0	17.07	14	-6.70	4.09	6.69	5.89	280.16	8.33	5.23
Myristoyl chloride	246.18	272.07	0.90	1	0	17.07	12	-5.82	3.82	5.78	-10.14	255.90	8.26	5.38

Table 2. ADMET Screening (**Absorption**)

compounds	caco2	MDCK	PAMPA	pgp_inh	pgp_sub	hia	f20	f30	f50
Oleic Acid	-5.08	-4.72	0.01	0.00	0.00	0.14	0.29	0.72	0.37
9-Octadecenoic acid	-5.08	-4.74	0.05	0.00	0.00	0.11	0.55	0.93	0.69
cis-Vaccenic acid	-5.08	-4.72	0.01	0.00	0.00	0.23	0.25	0.71	0.35
Palmitic acid	-5.10	-4.80	0.12	0.00	0.01	0.85	0.74	0.93	0.44
Ethyl Oleate	-5.02	-4.68	0.00	0.72	0.00	0.01	0.69	0.82	0.90
Paullinic acid	-5.08	-4.72	0.00	0.00	0.00	0.34	0.37	0.84	0.40
cis-11-Eicosenoic acid (Gonodoic acid)	-5.08	-4.72	0.01	0.00	0.00	0.15	0.37	0.78	0.34
9,12-Octadecadienoic acid (Linoleic acid)	-5.05	-4.84	0.02	0.00	0.00	0.01	0.15	0.39	0.61
9-Octadecenoic acid, methyl ester	-5.01	-4.72	0.00	0.35	0.01	0.02	0.66	0.94	0.92
Erucic acid	-5.09	-4.74	0.00	0.00	0.00	0.23	0.51	0.89	0.39
8,11-Eicosadienoic acid	-5.19	-4.89	0.06	0.00	0.00	0.00	0.08	0.25	0.51
10,13-Octadecadienoic acid	-5.06	-4.76	0.31	0.00	0.01	0.14	0.16	0.55	0.19
9-Octadecenal	-5.02	-4.74	0.00	0.47	0.00	0.07	0.78	0.98	0.86
2,3-Dihydroxypropyl elaidate	-5.10	-4.65	0.02	0.00	0.02	0.07	0.90	0.97	0.96
9-Octadecenoic acid (Z)-, 2,3-dihydroxypropyl ester	-5.07	-4.70	0.01	0.00	0.00	0.10	0.81	0.86	0.87
Palmitoyl chloride	-5.04	-4.83	0.00	0.00	0.03	0.72	0.82	0.97	0.89
Myristoyl chloride	-5.03	-4.81	0.00	0.01	0.03	0.59	0.71	0.92	0.83

Table 2. ADMET Screening (**Distribution**)

compounds	OATP1B1	OATP1B3	BCRP	BSEP	BBB	MRP1	PPB	logVDss	Fu
Oleic Acid	0.010916	0.995192	0.171241	0.960746	0.018328	0.995989	98.28009	-0.58651	0.536366
9-Octadecenoic acid	0.028162	0.999394	0.112075	0.996933	4.26E-05	0.83339	99.02585	-0.72022	0.275097
cis-Vaccenic acid	0.008857	0.992159	0.167983	0.962202	0.025631	0.993076	98.46742	-0.6162	0.471843
Palmitic acid	0.233934	0.999978	0.197781	0.092278	0.020998	0.989483	98.03125	0.592197	1.671825
Ethyl Oleate	0.015923	0.998591	0.509527	0.999972	0.003898	0.99836	98.57309	-0.46809	0.627767
Paullinic acid	0.012617	0.999268	0.22282	0.95503	0.0118	0.994882	98.33792	-0.43307	0.451716
cis-11-Eicosenoic acid (Gonodoic acid)	0.013367	0.998923	0.22195	0.967453	0.007895	0.997059	98.44711	-0.49326	0.367559
9,12-Octadecadienoic acid (Linoleic acid)	0.004594	0.556258	0.033971	0.985634	0.012275	0.999898	97.48871	-0.40317	1.573794

9-Octadecenoic acid, methyl ester	0.054164	0.99978	0.753108	0.999975	1.62E-05	0.910493	98.92529	-0.59133	0.409383
Erucic acid	0.019006	0.9999	0.288301	0.961237	0.003599	0.997828	98.38138	-0.28338	0.366547
8,11-Eicosadienoic acid	0.077603	0.995582	0.006611	0.998558	2.66E-06	0.973789	98.527	-0.44973	0.387182
10,13-Octadecadienoic acid	0.162649	0.985256	0.088073	0.297686	0.163442	0.979274	97.41384	-0.38448	1.752761
9-Octadecenal	0.092538	0.999885	0.778769	0.999903	9.07E-05	0.310665	98.94179	-0.35997	0.289403
2,3-Dihydroxypropyl elaidate	0.010949	0.999073	0.201523	0.999674	2.33E-05	0.603684	98.41618	-0.5667	0.665225
9-Octadecenoic acid (Z)-, 2,3-dihydroxypropyl ester	0.003624	0.989722	0.30687	0.995972	0.007479	0.988282	97.91246	-0.42756	1.157376
Palmitoyl chloride	0.482288	0.999998	0.855402	0.734978	0.047422	0.947632	100.0989	1.274957	0.672767
Myristoyl chloride	0.499568	0.999984	0.77656	0.712049	0.079735	0.927675	99.48834	1.064888	0.879993

Table 2. ADMET Screening (**Metabolism**)

compounds	CYP1A2-inh	CYP1A2-sub	CYP2C19-inh	CYP2C19-sub	CYP2C9-inh	CYP2C9-sub	CYP2D6-inh	CYP2D6-sub	CYP3A4-inh	CYP3A4-sub	CYP2B6-inh	CYP2B6-sub	CYP2C8-inh
Oleic Acid	0.997933	3.67E-08	0.998977	1.92E-05	0.010889	1	0.245062	0.980627	6.37E-05	1.04E-07	1	0.622782	0.999997
9-Octadecenoic acid	0.986833	2.65E-07	0.995456	6.23E-07	0.430375	0.999592	0.960561	0.999036	0.000476	2.09E-09	1	4.02E-05	1
cis-Vaccenic acid	0.839702	1.57E-06	0.748071	0.014808	0.33068	0.999986	0.470548	0.610624	0.000289	2.71E-06	0.999985	0.915919	0.999927
Palmitic acid	0.072816	8.23E-05	0.015061	0.061985	0.165551	0.99999	0.146891	0.732832	1.25E-05	5.50E-05	0.998758	0.999891	0.981206
Ethyl Oleate	1	1.93E-08	1	2.17E-06	0.210714	0.900269	0.267087	0.382839	0.994126	1.39E-05	1	0.001362	1
Paullinic acid	0.774472	1.69E-06	0.588776	0.034886	0.429223	0.999999	0.499908	0.930497	0.000142	9.34E-07	0.999994	0.993446	0.999977
cis-11-Eicosenoic acid (Gonodoic acid)	0.998086	2.20E-07	0.99822	2.20E-05	0.002398	0.999999	0.437853	0.932628	5.00E-05	6.24E-07	1	0.222002	0.999993
9,12-Octadecadienoic acid (Linoleic acid)	0.498693	6.45E-08	0.563965	0.032732	0.712985	1	0.008972	0.960748	0.000895	4.66E-08	1	1	0.999998
9-Octadecenoic acid, methyl ester	0.999999	2.99E-05	1	2.59E-06	0.973801	0.286273	0.948511	0.999986	0.99931	5.68E-09	1	3.33E-07	1
Erucic acid	0.997083	2.37E-07	0.996316	5.29E-05	0.003645	1	0.466972	0.991609	2.47E-05	2.15E-07	1	0.798807	0.999998
8,11-Eicosadienoic acid	0.9994	1.77E-06	0.997205	2.94E-06	0.602963	0.999997	0.603564	0.996223	7.19E-05	2.28E-09	1	0.109827	1

10,13-Octadecadienoic acid	0.965724	1.50E-06	0.100526	0.406286	0.382513	1	0.008344	0.957546	0.000142	2.19E-07	0.999632	0.99998	0.999849
9-Octadecenal	0.999999	2.49E-05	1	2.89E-07	0.329084	0.559202	0.995868	0.999926	0.996972	5.04E-07	1	3.64E-09	1
2,3-Dihydroxypropyl elaidate	0.999855	4.53E-07	0.999888	3.45E-08	0.015521	0.055085	0.892864	0.999281	0.997432	1.11E-07	1	5.19E-15	1
9-Octadecenoic acid (Z)-, 2,3-dihydroxypropyl ester	0.999984	6.16E-08	0.999969	7.21E-07	0.000101	0.99284	0.15895	0.98777	0.988177	5.97E-06	1	1.05E-10	0.999997
Palmitoyl chloride	0.983825	0.009888	0.9897	0.067105	0.765154	0.991999	0.691863	0.898322	0.066963	0.000788	0.998283	0.97735	0.994314
Myristoyl chloride	0.983476	0.009267	0.989251	0.065107	0.765634	0.990646	0.680733	0.888856	0.066557	0.000889	0.997856	0.974985	0.992994

Table 2. ADMET Screening (**Excretion**)

compounds	LM-human	cl-plasma	t0.5	BCF
Oleic Acid	0.000149	3.49975	0.75405	0.79806
9-Octadecenoic acid	0.001552	3.432648	0.852094	0.727181
cis-Vaccenic acid	0.000583	3.552657	0.777839	0.807516
Palmitic acid	0.000682	3.770445	0.932167	0.810951
Ethyl Oleate	0.39716	5.502497	0.328008	1.18135
Paullinic acid	0.000279	3.710837	0.90164	0.676626
cis-11-Eicosenoic acid (Gonodoic acid)	0.000106	3.657665	0.814508	0.659176
9,12-Octadecadienoic acid (Linoleic acid)	0.003762	3.964195	0.501936	0.96446
9-Octadecenoic acid, methyl ester	0.981261	5.314329	0.412084	1.098244
Erucic acid	5.06E-05	3.758287	0.978199	0.546136
8,11-Eicosadienoic acid	0.000842	3.669612	0.742325	0.720842
10,13-Octadecadienoic acid	0.058754	3.84482	0.602291	1.014105
9-Octadecenal	0.099715	5.359458	0.44715	1.292334
2,3-Dihydroxypropyl elaidate	0.387584	4.830682	0.650858	1.185626
9-Octadecenoic acid (Z)-, 2,3-dihydroxypropyl ester	0.040961	4.875492	0.59957	1.196522

Palmitoyl chloride	0.158053	5.266185	0.650291	0.921462
Myristoyl chloride	0.15834	5.379245	0.487284	1.103025

Table 2: ADMET screening (Toxicity)

DILI	Ames	ROA	FDAMDD	SkinSen	Carcinogenicity	EC	EI	Respiratory	H-HT	Neurotoxicity-DI	Ototoxicity	Hematotoxicity	Nephrotoxicity-DI	Genotoxicity
0.009204	0.088136	0.080827	0.21532	0.998412	0.135301	0.995462	0.995975	0.789521	0.25589	0.009401	0.260278	0.057138	0.389779	3.38E-06
0.043036	0.015045	0.041973	0.220525	0.991537	0.093645	0.819646	0.991543	0.929614	0.679709	0.028612	0.356216	0.180869	0.274662	6.49E-05
0.007315	0.072616	0.077464	0.188716	0.998267	0.102718	0.996518	0.996437	0.804718	0.236883	0.007757	0.265451	0.048265	0.39998	1.51E-06
0.187545	0.04709	0.124449	0.178303	0.908553	0.269677	0.98317	0.997497	0.932895	0.422959	0.029615	0.298118	0.271056	0.236295	4.88E-06
0.006905	0.093331	0.052253	0.147851	0.99939	0.215256	0.996676	0.996446	0.56017	0.157206	0.006205	0.101974	0.024739	0.247841	1.31E-06
0.006838	0.054738	0.071679	0.199485	0.998931	0.092427	0.996464	0.99652	0.855738	0.241303	0.005091	0.252369	0.043632	0.370478	3.69E-07
0.008607	0.067208	0.075256	0.226972	0.999021	0.122592	0.995399	0.996042	0.84396	0.261186	0.006206	0.247835	0.051953	0.361916	8.39E-07
0.000428	0.106712	0.032535	0.034884	0.999862	0.021914	0.999086	0.998675	0.799486	0.137747	0.005296	0.207088	0.010493	0.378262	1.20E-06
0.032593	0.028208	0.050731	0.273823	0.996948	0.200859	0.887128	0.986293	0.879044	0.688824	0.047038	0.229713	0.145645	0.376174	2.96E-05
0.008047	0.050589	0.069624	0.239281	0.999396	0.110556	0.995327	0.996133	0.886177	0.265902	0.00407	0.235342	0.046983	0.33366	2.05E-07
0.274641	0.005822	0.055482	0.228222	0.993859	0.09344	0.006342	0.881163	0.964417	0.899835	0.089319	0.685984	0.38957	0.319562	0.001658
0.003228	0.132184	0.06295	0.071064	0.999552	0.113212	0.997237	0.998581	0.704964	0.10828	0.009856	0.227768	0.024697	0.218648	1.45E-06
0.064397	0.017779	0.059394	0.157669	0.999275	0.029726	0.977923	0.994646	0.95095	0.622978	0.050897	0.186168	0.115256	0.342632	0.000138
0.006745	0.085857	0.006776	0.090301	0.999324	0.150585	0.035465	0.73404	0.206078	0.555285	0.015886	0.725273	0.054477	0.390433	1.51E-05
0.001396	0.373738	0.013592	0.087603	0.999875	0.21119	0.642207	0.852805	0.068943	0.168684	0.005153	0.627084	0.015631	0.522109	7.72E-07
0.019241	0.692585	0.609603	0.707805	0.99867	0.531424	0.999999	0.999857	0.999999	0.420835	0.163365	0.280538	0.746177	0.0524	1.54E-05
0.020565	0.752865	0.629369	0.693362	0.997844	0.56041	0.999999	0.999854	0.999999	0.414925	0.229783	0.294505	0.765685	0.058944	6.28E-05

Table 3: Principal component accounted for most of variance.

	PC 1	PC 2	PC 3	PC 4	PC 5	PC 6	PC 7	PC 8	PC 9	PC 10	PC 11	PC 12	PC 13	PC 14	PC 15	PC 16
Oleic Acid	-1.639	-0.250	0.286	-0.466	0.073	0.229	0.031	0.091	-0.059	0.170	-0.003	-0.137	-0.269	0.169	0.081	-0.061
9-Octadecenoic acid	-1.266	-0.357	1.394	0.247	-0.099	-0.258	-0.067	-0.037	-0.527	0.424	-0.060	0.058	0.171	-0.041	0.054	0.002
cis-Vaccenic acid	-1.594	-0.022	0.265	-0.430	0.275	0.007	-0.193	0.054	0.062	-0.021	-0.384	0.222	-0.167	0.000	-0.051	0.097
Palmitic acid	-1.613	1.023	-1.400	0.005	-0.564	-0.982	0.066	-0.378	-0.157	-0.161	0.058	-0.013	-0.063	0.015	0.009	0.004
Ethyl Oleate	2.895	-0.671	-0.580	-0.610	0.653	0.165	-0.088	-0.659	-0.075	-0.035	-0.253	-0.110	0.077	-0.029	0.029	-0.021
Paullinic acid	-1.604	0.181	0.210	-0.420	0.193	-0.147	-0.079	0.220	0.217	-0.256	-0.084	0.243	0.130	-0.051	0.010	-0.114
cis-11-Eicosenoic acid (Gonodoic acid)	-1.558	-0.135	0.641	-0.628	0.008	0.110	0.080	-0.129	0.005	0.093	0.245	-0.173	-0.042	-0.096	-0.194	-0.014
9,12-Octadecadienoic acid (Linoleic acid)	-1.321	-0.560	-1.187	0.693	0.543	0.356	-0.089	0.404	-0.434	-0.351	-0.017	-0.120	0.041	-0.005	-0.029	0.007
9-Octadecenoic acid, methyl ester	2.469	-0.502	0.435	0.180	0.552	-0.545	-0.801	0.144	0.113	-0.004	0.335	0.032	-0.043	0.045	0.000	0.016
Erucic acid	-1.513	0.187	0.495	-0.667	0.003	0.041	0.193	0.128	0.270	-0.203	0.141	-0.186	0.189	-0.005	0.121	0.076
8,11-Eicosadienoic acid	-1.148	-0.665	1.068	1.234	-0.339	0.418	-0.041	-0.554	0.203	-0.259	0.057	0.050	-0.045	0.015	0.012	-0.001
10,13-Octadecadienoic acid	-1.357	-0.453	-1.602	0.619	0.312	0.053	0.124	0.024	0.416	0.536	0.007	0.014	0.088	0.002	-0.008	0.003
9-Octadecenal	2.539	-0.543	0.581	0.294	0.439	-0.399	1.037	0.164	-0.006	-0.067	0.041	0.074	-0.081	0.008	-0.013	0.005
2,3-Dihydroxypropyl elaidate	2.053	-1.289	0.080	0.147	-1.108	-0.204	-0.167	0.363	0.154	-0.009	-0.312	-0.182	0.000	-0.032	-0.024	-0.013
9-Octadecenoic acid (Z)-, 2,3-dihydroxypropyl ester	1.589	-0.905	-0.933	-0.619	-0.775	0.586	0.068	0.005	-0.178	0.026	0.285	0.260	0.026	0.027	0.009	0.019
Palmitoyl chloride	1.617	2.658	0.337	0.168	-0.125	0.230	0.001	0.036	-0.001	0.023	-0.098	-0.013	0.168	0.166	-0.086	0.001
Myristoyl chloride	1.452	2.303	-0.091	0.254	-0.040	0.340	-0.076	0.122	-0.003	0.095	0.042	-0.018	-0.180	-0.188	0.080	-0.006

Table 4: Top ten predicted hub genes for FA and CVD-related disease.

TOP 10 HUB Genes	
1	SRC
2	PIK3CA
3	STAT3
4	PIC3CB
5	PIC3CD
6	AKT1
7	MAPK1
8	GRB2
9	PTPN11
10	HSP90AA1

Table 5: Detail average values of plant attributes and fatty acid composition.

Parameter	NARC-Sarson				CanolaSuper8				Punjab Canola				Sundal Canola			
	Gy-0	Gy-10	Gy-20	Gy-30	Gy-0	Gy-10	Gy-20	Gy-30	Gy-0	Gy-10	Gy-20	Gy-30	Gy-0	Gy-10	Gy-20	Gy-30
Plant Height	216.47	176.6	164.6	172.2	214.6	185.9	174.2	161	185.6	176.2	176.9	161.3	193.9	186.7	161.6	147.3
Root Length	15.9	16.1	20.4	23.2	19.1	19.2	16.3	19.7	22.93	23.8	20.5	19.3	15.1	20.6	15.2	16.7
Pods Number	125.6	163.3	148	156.3	102.3	118	147	141	171	119.6	113.3	107	144.6	124	85.3	109.6
100 Seed Weight	0.42	0.34	0.37	0.34	0.39	0.38	0.32	0.36	0.38	0.37	0.42	0.37	0.43	0.38	0.35	0.35
Oil Content (%)	39.8	45.8	45.8	39.7	39.1	43.6	41.8	48.2	45.2	48.2	48.9	44.3	42.2	39.1	45.6	47.8
Protein Content (%)	23.5	24.3	25.2	22.4	24.7	25.2	27.3	30.5	21.5	26.4	24.2	26.1	20.2	25.4	22.3	24.4
Moisture Content (%)	5.6	4.9	5.7	5.2	6.2	4.9	5.7	5.1	4.8	5.2	4.9	5.6	5.8	4.9	5.1	5.7
Oleic Acid (%)	50.8	53.3	55.7	57.1	54.3	51.4	60.6	51.6	50.2	54.2	54.6	51.2	51.2	55.4	55.6	52.2
Linolenic Acid (%)	11.1	10.3	8.3	7.6	8.3	9.9	9.2	9.2	7.9	8.4	8.9	9.6	9.7	8.2	9.4	9.4
Palmitic Acid (%)	9.1	9.4	9.9	8.7	9.6	8.2	7.8	8.5	9.1	8.7	7.5	8.3	7.9	8.9	9.1	8.4
